# Targeting Smad3 Phosphorylation Attenuates Anastomotic Intimal Hyperplasia and Perigraft Fibrosis in Decellularized Tissue-Engineered Vascular Grafts

**DOI:** 10.34133/bmr.0241

**Published:** 2025-10-17

**Authors:** Peng Lu, Tun Wang, Sheng Liao, Zhenyu He, Siyuan Cheng, Tianjian Wang, Zibo Cheng, Yangyang An, Sirui Zhou, Mo Wang, Qian Zhang, Chang Shu

**Affiliations:** ^1^ Department of Vascular Surgery, The Second Xiangya Hospital, Central South University, Changsha 410011, China.; ^2^ Institute of Vascular Diseases, Central South University, Changsha 410011, China.; ^3^ State Key Laboratory of New Textile Materials and Advanced Processing Technologies, Wuhan Textile University, Wuhan 430200, China.; ^4^ Center of Vascular Surgery, Fuwai Hospital, Chinese Academy of Medical Sciences and Peking Union Medical College, Beijing 100037, China.

## Abstract

Traditional polymer-based arteriovenous grafts (AVGs) for hemodialysis access suffer from poor long-term patency, high reintervention rates, and susceptibility to infection. In contrast, decellularized tissue-engineered vascular grafts (dTEVGs) demonstrate improved patency, long-term durability, and resistance to infection. However, vascular stenosis and occlusion caused by anastomotic intimal hyperplasia (AIH), as well as vascular stiffening and calcification from excessive perigraft fibrosis (PGF), remain major challenges in the clinical use of dTEVGs for AVGs. M2 macrophage infiltration plays a key role in the biological processes of pro-regeneration and the clinical application of dTEVGs. However, in elastin-rich dTEVGs commonly used clinically, the elastic fiber layers form a barrier to cell infiltration, potentially limiting their biological functions. Therefore, the specific impact of M2 macrophage infiltration on dTEVGs in AVGs remains unclear. Through parallel analysis of human explants and a rat dTEVG-AVG model, we found that M2 macrophage infiltration predominates in dTEVGs, and this infiltration is associated with AIH and PGF. Furthermore, IL-4-loaded poly(lactic-*co*-glycolic acid)/gelatin methacryloyl delivery systems selectively enhanced M2 macrophage polarization, while sustained M2 macrophage infiltration triggered TGF-β1/Smad3-dependent myofibroblast activation, leading to increased AIH and PGF. Pharmacological inhibition of Smad3 phosphorylation selectively alleviated AIH and PGF without affecting M2 macrophage recruitment or other associated biological functions. These findings reveal the dual role of M2 macrophages in dTEVGs for AVGs, which, while promoting pro-regeneration, unexpectedly accelerate AIH and PGF. A targeted Smad3 inhibition strategy selectively alleviates AIH and PGF caused by M2 macrophage infiltration, without compromising M2 macrophage-associated functions.

## Introduction

Chronic kidney disease is a progressive condition leading to kidney failure and premature death [1], with arteriovenous grafts (AVGs) serving as a critical lifeline for end-stage renal disease patients requiring hemodialysis [2]. Traditional polymer AVG materials, such as expanded polytetrafluoroethylene, lack biological activity and are not suitable for long-term use due to poor long-term patency, high reintervention rates, and susceptibility to infection [3,4]. Decellularized tissue-engineered vascular grafts (dTEVGs) demonstrate improved patency, long-term durability, and resistance to infection, thereby providing a superior alternative [5,6].

Anastomotic intimal hyperplasia (AIH) and perigraft fibrosis (PGF) are major challenges hindering the clinical use of dTEVGs [6–9]. AIH critically compromises graft patency and reduces hemodialysis access flow through vascular stenosis and occlusion, ultimately diminishing the efficacy of AVGs [4,10]. Currently, AIH is considered the major pathological driver of late graft dysfunction in long-term dialysis access [2]. PGF emerges as a pathological hallmark across cardiovascular disorders, where physiological adventitial matrix homeostasis becomes disrupted under inflammatory or immune-challenged states, which leads to vascular stiffening and calcification [9,11,12]. This dysregulation manifests as excessive collagen deposition, inducing vascular stiffening and compliance loss, and finally contribute to the adverse outcome of dTEVGs in AVGs [11]. Nevertheless, due to the limitations of animal models, research addressing AIH and PGF in dTEVGs for AVGs remains limited.

Innate immune responses mediate the postimplantation integration of biomaterials [12–14]. Our preliminary investigations revealed predominant M2 macrophage infiltration within dTEVGs from both clinical specimens and rat models [15]. Interestingly, however, all dTEVGs currently used clinically are elastin-rich, which hinders cell infiltration and associated biological functions [7]. Therefore, how M2 macrophage infiltration influences elastin-rich dTEVGs, particularly its relationship with AIH and PGF, remains unclear.

In this study, through integrated human and novel rat AVG models, we reveal the dual role of M2 macrophages: while they contribute to the pro-regenerative process, they also promote TGF-β1/Smad3-driven AIH and PGF. By targeting Smad3 phosphorylation, we achieved selective mitigation of AIH and PGF while preserving M2 macrophage recruitment and its associated biological functions, establishing a precision strategy to optimize dTEVGs for AVGs in hemodialysis.

## Materials and Methods

### Acquisition of dTEVG specimens

The acquisition of all tissues was approved by the Medical Ethics Committee of the Second Xiangya Hospital (Approval No. 2019-Clinical Research-82), and patients provided informed consent for the use of the samples. The dTEVG samples implanted in humans were immediately rinsed in phosphate-buffered saline (PBS) and then immersed in 4% paraformaldehyde (PFA) for fixation as soon as the tissue was removed. The tissue was then dehydrated in ethanol and xylene and embedded in paraffin. Native iliac artery specimens were collected from patients undergoing amputation procedures and immediately cryopreserved.

### Decellularization of the native iliac artery

Decellularization of the native iliac artery was performed as described previously [16]. Briefly, the iliac artery was incubated for 22 h in 3-[(3-chloamidopropyl)dimethyl-ammonium]-1-propanesulfonate (CHAPS) buffer (8 mM CHAPS [Sigma-Aldrich, St Louis, MO], 1 M NaCl [Sigma-Aldrich], and 25 mM ethylenediaminetetraacetic acid (EDTA) [Sigma-Aldrich] in PBS), followed by 22 h in sodium dodecyl sulfate (SDS) buffer containing 1.8 mM SDS (Sigma-Aldrich), 1 M NaCl, and 25 mM EDTA in PBS and 48 h of PBS washes. We further subjected the decellularized vascular grafts to DNase I (3,000 U/ml) and RNase A (3,000 U/ml) treatment for 1 h. The decellularization process was performed at room temperature. The decellularized iliac artery was stored in PBS containing penicillin (100 U/ml) and streptomycin (0.1 mg/ml) and placed at 4 °C until use.

### Decellularization of rat carotid arteries and construction of a rat dTEVG-AVG model

All experiments were approved by the Animal Care and Use Committee of the Second Xiangya Hospital of Central South University (Approval No. 20231043). All experiments were performed in accordance with the National Institutes of Health Guide for the Care and Use of Laboratory Animals. Male Sprague Dawley rats (9 weeks old) were dissected to retrieve the carotid artery (CA) (1.5 cm). The CAs were frozen at −20 °C, thawed, and incubated in CHAPS buffer (8 mM CHAPS, 1 M NaCl, and 25 mM EDTA in PBS) for 24 h, followed by incubation in SDS buffer (1.8 mM SDS, 1 M NaCl, and 25 mM EDTA in PBS) for 24 h. The CAs were washed in PBS for 48 h and then treated with DNase I (3,000 U/ml) and RNase A (3,000 U/ml) for 1 h followed by PBS washing. The decellularization process was performed at room temperature. After completing the decellularization process, the decellularized rat CAs were stored in PBS containing penicillin (100 U/ml) and streptomycin (0.1 mg/ml) at 4 °C until use. The construction of the dTEVG-AVG rat model was performed following previous research [15]. We recommend having at least one trained surgeon perform the surgical procedures. CHAPS and SDS were purchased from Sigma-Aldrich (Darmstadt, Germany). EDTA, DNase I, and RNase A were purchased from BioFroxx (Germany).

For intervention, an IL-4 nanoparticle (NP)-gelatin methacryloyl (GelMA) hydrogel was placed near the dTEVG area in the rats’ neck region to ensure that the released IL-4 could penetrate the dTEVG area without affecting the migration of the surrounding tissue cells to the dTEVG. SIS3 (2 mg/kg, MedChemExpress, Shanghai, China) was administered continuously via intraperitoneal injection for 21 d. All animals were randomly assigned to groups.

### Tissue paraffin embedding and sectioning

After collection, specimens were fixed in 4% PFA for 24 h. Following fixation, tissues were dehydrated through sequential immersion in 75% ethanol and absolute ethanol. The dehydrated tissues were then cleared in xylene. The tissues were embedded in paraffin after immersion in melted paraffin in a constant-temperature oven. Once the paraffin blocks were fully cooled and solidified, sections were cut using a microtome to a thickness of approximately 4 μm. The slides were baked at 60 °C overnight and stored at room temperature for future use.

### Hematoxylin and eosin staining

Hematoxylin and eosin (HE) staining was performed using an HE staining kit (Servicebio, China, G1005). Tissue sections were deparaffinized in xylene and rehydrated through a series of ethanol baths. Sections were stained with hematoxylin for 3 min and then washed to remove excess stain. The sections were differentiated in an acidic ethanol solution for 1 min and washed with distilled water. Finally, the sections were stained with eosin for 30 s and washed to remove excess stain. After staining, sections were dehydrated in 75% and 95% ethanol, cleared in xylene, and mounted with resin.

### Masson staining

Masson staining was performed using a Masson’s trichrome stain kit (Solarbio, China, G1346). Tissue sections were deparaffinized in xylene and rehydrated through graded ethanol. Sections were stained with Lichun Red-Magenta solution for 5 to 10 min, differentiated in weak acid solution for 30 s, and treated with phosphomolybdic acid for 1 to 2 min. Sections were then differentiated in weak acid solution for 30 s and stained with aniline blue for 1 to 2 min, followed by further differentiation. Sections were quickly dehydrated in 95% ethanol for 2 to 3 s and in absolute ethanol twice for 5 to 10 s each and cleared in xylene twice for 1 to 2 min each. Sections were mounted with neutral resin.

### Elastin–van Gieson staining

Elastin–van Gieson (EVG) staining was performed using a kit (Haoke Biotechnology, China, HKT2034). Tissue sections were deparaffinized and rehydrated through graded ethanol. EVG staining solution was prepared by mixing EVG staining solutions A, B, and C in a ratio of 5:2:2. Sections were stained with the EVG solution for 5 min and then washed with tap water. EVG solution B was diluted 5 times, used for differentiation, and washed with tap water, controlling the differentiation degree under a microscope until elastic fibers were purple-black and the background was gray-white to nearly colorless. Sections were then stained with EVG solution D for 1 to 3 min, quickly washed with water, dehydrated in absolute ethanol, cleared in xylene, and mounted with neutral resin. Microscopic examination and image analysis were performed.

### Sirius Red staining

Sirius Red staining was performed using a kit (Solarbio, China, G1472). Tissue sections were deparaffinized and rehydrated. Sirius Red staining solution was prepared fresh before use. The sections were stained with Sirius Red staining solution for 15 min and rinsed with running water to remove excess dye. The sections were dehydrated through a series of ethanol concentrations starting from 75%, cleared in xylene, and mounted with neutral resin. Polarized light microscopy was used for imaging.

### Immunohistochemistry

Tissue sections were deparaffinized using xylene and a graded series of alcohols. Sections were heated in 0.01 M citric acid buffer (pH 6.0) at 100 °C for 20 min and cooled to room temperature. After washing 3 times with PBS, sections were blocked with 5% normal goat serum in PBS (pH 7.4) for 1 h at room temperature. Sections were then incubated at 4 °C overnight. The primary antibodies are as follows: CD3 (Proteintech, 17617-1-AP, 1:200), CD68 (Proteintech, 66231-2-Ig, 1:1,000), CD206 (Abcam, ab64693, 1:200), and iNOS (Abcam, ab178945, 1:50).

### Immunofluorescence

For tissues, deparaffinized tissue sections were rehydrated through graded ethanol. Sections were heated in Tris–EDTA buffer (pH 9.0) at 100 °C for 20 min and then cooled to room temperature. Sections were blocked with 10% goat serum at room temperature for 1 h. Primary antibodies were incubated overnight at 4 °C. After warming to room temperature, secondary antibodies were incubated for 1 h. Nuclei were stained with 4′,6-diamidino-2-phenylindole (DAPI). Immunofluorescence (IF) microscopy was used for observation.

For cells, cells were seeded in culture dishes to achieve an appropriate density before staining. The medium was gently removed followed by PBS washing. Then, cells were fixed with 4% PFA for 15 min followed by PBS washing to remove excess fixative. Permeabilization solution (0.1% Triton X-100) was added, and cells were incubated for 15 min. Afterward, 5% bovine serum albumin was used to block nonspecific binding sites for 1 h. Primary antibodies were incubated overnight at 4 °C. After warming to room temperature and washing 3 times with PBS, fluorescence-labeled secondary antibodies were added and incubated for 1 h. Cells were washed 3 times with PBS and then stained with DAPI before mounting. Results were observed under a fluorescence microscope.

The primary antibodies are as follows: CD206 (Abcam, ab64693, 1:200), CD68 (Abcam, ab31630, 1:200), CD68 (Servicebio, GB113109, 1:200), iNOS (Abcam, ab178945, 1:50), α-SMA (Proteintech, 67735-1-Ig, 1:200), collagen I (Cell Signaling, 72026, 1:200), collagen I (Servicebio, GB11022, 1:100), phalloidin (Yeasen, 40735ES80, 1:200), CD3 (Proteintech, 17617-1-AP, 1:200), IL-10 (Servicebio, GB11108), and TGF-β1 (Abcam, ab215715).

### Preparation and characterization of PLGA NPs

Poly(lactic-*co*-glycolic acid) (PLGA) NPs were prepared using a nanoprecipitation method. The organic phase containing dissolved PLGA (Sigma-Aldrich, Darmstadt, Germany) and IL-4 (MedChemExpress, Shanghai, China) was slowly injected into the aqueous phase containing dissolved polyvinyl alcohol at a volume ratio of 1:10. The mixture was stirred uniformly at room temperature under an ice bath for 30 min, followed by removal of acetone through rotary evaporation at room temperature for 30 min to obtain the NP suspension. The liquid was transferred into a 10-kDa ultrafiltration centrifuge tube and centrifuged at 2,500 g for 30 min to remove polyvinyl alcohol (Aladdin, Shanghai, China) and some small water-soluble impurities. The NP precipitate was then resuspended in pure water or PBS. NPs were lyophilized using a freeze dryer. The particle size and zeta potential of PLGA NPs and membrane-coated NPs were measured using a dynamic light scattering particle size analyzer. The morphological characteristics of PLGA NPs were observed using a transmission electron microscope.

### Drug loading and encapsulation efficiency of NPs

First, a specific amount of freeze-dried IL-4@PLGA NP powder was accurately weighed, suspended in 10% dimethyl sulfoxide, and mixed with vortexing followed by a brief warm bath. Next, the suspension was quickly diluted with PBS to reduce the dimethyl sulfoxide concentration to below 1%. An IL-4 enzyme-linked immunosorbent assay (ELISA) kit was used to measure the IL-4 content. Finally, the drug loading capacity was calculated using the following formula: drug loading capacity = (amount of drug in NPs/total weight of NPs) × 100%. The drug loading capacity of the NPs was calculated to be approximately 3.98% ± 0.02%. The encapsulation efficiency was calculated using the following formula: encapsulation efficiency = (amount of drug encapsulated in NPs/amount of drug added) × 100%. The encapsulation efficiency of the NPs was found to be 62.47% ± 0.14%.

### Preparation and photo-cross-linking of GelMA hydrogel

GelMA hydrogel was purchased from Engineering for Life (Suzhou, China) and prepared according to specific steps. First, an appropriate amount of PBS/drug was added to GelMA, and the mixture was magnetically stirred at 37 °C in the dark for 1 h to prepare the porous GelMA hydrogel precursor solution. The solution was then filtered using a 0.22-μm sterile syringe filter and stored at 37 °C in the dark. For the in vitro experiment, 100 μl of the gel was irradiated with a 405-nm light source for 15 s to cross-link the hydrogel. For the in vivo experiment, 500 μl of the GelMA hydrogel was applied around the dTEVG after model construction and then photo-cross-linked.

### Scanning electron microscopy evaluation of gel and embedded nanoparticles

To observe the microstructure of the gel and embedded NPs, scanning electron microscopy (SEM) was utilized. After photo-cross-linking of the gels containing or not containing NPs, they were freeze-dried using a freeze dryer. The dried samples were then sectioned into appropriate sizes. The surfaces of the dried samples were coated with gold. The SEM parameters, such as accelerating voltage, working distance, and scan speed, were adjusted to obtain clear images. During observation, the magnification was adjusted as needed to capture the structural information of the vascular tissue at different scales. Specialized image processing software was used for subsequent analysis and measurements of the images.

### Drug release curve measurement

In vitro drug release studies were conducted using PBS with a pH of 7.4. Briefly, 100 μl of gel containing drug-loaded NPs was placed in 1 ml of PBS at 37 °C. From day 0, 100 μl of the supernatant was taken and frozen at −80 °C for detection and replaced with 100 μl of fresh PBS to continue the incubation at 37 °C. The drug content was measured using an IL-4 ELISA kit (ml107056, Shanghai Enzyme-linked Biotechnology Co., Ltd., China). The release kinetics of IL-4 from the hydrogel were monitored over a 21-d period. Sampling was performed at 6 time points: days 1, 3, 5, 7, 14, and 21.

### Enzyme-linked immunosorbent assay

Cell culture supernatants were centrifuged at 1,000 g for 15 min at 2 to 8 °C, and the supernatants were either tested directly or aliquoted and stored at −80 °C to avoid repeated freeze–thaw cycles. For standard preparation, five 1.5-ml centrifuge tubes were used, each containing 150 μl of sample diluent, followed by serial dilutions. On an ELISA plate, blank wells and sample wells were set up by adding 40 μl of sample diluent and 10 μl of test samples, with a final dilution factor of 5×. The plate was mixed, sealed, and incubated at 37 °C for 30 min. After incubation, the plate was washed by removing the seal, discarding the liquid, and adding washing buffer, allowing it to sit for 30 s before discarding, repeating this process 5 times, and then drying. Standard curves were generated using the “Curve Expert” software, and sample concentrations were calculated. If the samples were diluted, the actual concentration was determined by multiplying by the dilution factor. The TGF-β1, IL-10, and TNFα ELISA kits were bought from Shanghai Enzyme-linked Biotechnology Co., Ltd., China.

### Cell experiments

#### Bone-marrow-derived macrophages

Macrophages were isolated from the bone marrow of 3-week-old male Sprague Dawley rats or 8-week-old C57 mice. After euthanizing the rats or mice, the femurs and tibias were collected. The bone marrow cavities were repeatedly flushed with Dulbecco’s modified Eagle’s medium (DMEM) (Gibco, California, USA) containing 10% fetal bovine serum (FBS). The bone marrow was mixed thoroughly and filtered using a 40-μm filter. The cells were centrifuged, and the red blood cells were lysed. The resulting cells were seeded in DMEM containing 10% FBS and 20 ng/ml macrophage colony-stimulating factor (MedChemExpress, Shanghai, China) and stimulated continuously for 7 d to obtain bone-marrow-derived macrophages (BMDMs). The cell suspension was cultured at 37 °C with 5% CO_2_. On the third day, the medium was replaced to remove nonadherent cells and refresh the medium, with subsequent medium changes every 3 d.

#### Regulation of macrophage polarization by IL-4 NP-GelMA hydrogel

After continuous IL-4 release from IL-4 NP-GelMA hydrogel for 14 d, the gel was removed, washed with sterile PBS, and placed in complete medium with 10% FBS at 37 °C for 24 h. The medium was then collected for subsequent cell culture. The BMDMs of rats were cultured in the above medium, with the control group using ordinary medium. After 24 h, the cell supernatant was collected for ELISA detection; cells were washed twice with PBS and lysed with radioimmunoprecipitation assay (RIPA) buffer for protein extraction; for IF detection, cells were fixed with 4% PFA for 15 min.

#### M2 macrophage and fibroblast co-culture

(a) Mouse BMDMs were successfully cultured in a transwell chamber, and IL-4 NP-GelMA hydrogel was used to polarize BMDMs into M2 macrophages. (b) NIH 3T3 cells were cultured in a 24-well plate to 60% to 70% confluence using DMEM containing 10% FBS at 37 °C with 5% CO_2_. NIH 3T3 cells underwent starvation before co-culturing. (c) The Transwell chamber with M2 macrophages was placed into the 24-well plate containing NIH 3T3 cells for co-culture. In inhibition experiments, NIH 3T3 cells were co-treated with LY2109761 (1 and 10 μM) or SIS3 (1 and 10 μM) and then co-cultured with M2 macrophages for 12 h. Cells were washed twice with PBS and lysed with RIPA buffer for protein extraction; for IF, cells were fixed with 4% PFA for 15 min. SIS3 and LY2109761 were purchased from MedChemExpress (Shanghai, China).

### Western blot

Total protein was extracted using RIPA lysis buffer, and protein concentrations were assessed using the colorimetric method (Bio-Rad, Hercules, CA). For Western blot, proteins (20 to 40 μg) were separated on 8% to 10% SDS–polyacrylamide gel electrophoresis gels and transferred to polyvinylidene fluoride membranes (0.45-μm pore size; Immobilon, Millipore). The membranes were blocked with 5% bovine serum albumin at room temperature for 1 h and then incubated overnight at 4 °C with primary antibodies. Afterward, the membranes were incubated with horseradish peroxidase-conjugated secondary antibodies for 1 h at room temperature. The blots were developed using the Western Lightning Plus ECL reagent. If necessary, stripping buffer was used to elute antibodies, and the membranes were reprocessed as described.

The primary antibodies were as follows: Arg-1 (Proteintech, 16001-1-AP, 1:1,000), GAPDH (Proteintech, 10494-1-AP, 1:5,000), collagen I (Servicebio, GB11022, 1:1,000), α-SMA (Proteintech, 14395-1-AP, 1:1,000), p-Smad3 (Cell Signaling, #9520, 1:1,000), and Smad3 (Cell Signaling, #9523, 1:1,000).

### Flow cytometry

We followed a similar protocol as previously described [17]. Briefly, for isolation of single cells, dTEVG samples after 21-d implantation were collected. To avoid and limit blood contamination, we transcardially perfused the rat with PBS, followed by grafts harvested from the rat. After 3 washes with PBS, grafts were cut into fine pieces and digested in digestion solutions at 37 °C with reciprocal shaking for a total of 40 min. Detached cells were then collected through a 40-μm cell strainer and suspended in DMEM with 10% FBS (Gibco, 10270). Cells were further stained with Zombie Aqua Fixable Viability Kit (BioLegend, 423101). Surface staining was performed with anti-CD86-FITC (BioLegend, 200305; 1:100) and anti-CD68-PE (BioLegend, 201004; 1:100) for 15 min at 4 °C. Cells were then fixed and permeabilized with BD Cytofix/Cytoperm Solution (#554714) for 20 min at 4 °C. Then, staining with anti-CD206 (Abcam, 64693, 1:100) for 30 min at 4 °C, after 2 washes, secondary antibodies (Alexa Fluor 594, Abcam, ab150168) were applied for 30 min at 4 °C. After washing, cells were resuspended. Analysis was performed by BD FACSAria II Flow Cytometer (BD Biosciences).

### Statistical analysis

Image analysis was performed using the ImageJ software. For IF and immunohistochemical staining, we quantified the number of positive cells and calculated the average optical density based on the specific marker and experimental objective. For Masson’s trichrome staining, the collagen area was measured. EVG staining was used to identify the elastic lamina, and the thickness of neointimal hyperplasia was quantified accordingly.

Data are expressed as mean ± standard error of the mean. All data were analyzed using the Prism 8 software (GraphPad Software, Inc., La Jolla, CA). Different statistical tests were chosen based on data characteristics. For comparisons between 2 groups, an unpaired *t* test was used if the data were normally distributed with equal variance, Welch’s *t* test was used for normally distributed data with unequal variance, and Mann–Whitney *U* test was used for nonnormally distributed data. For comparisons involving 2 factors and multiple groups, 2-way analysis of variance was used if the data were normally distributed with equal variance. Post hoc testing was performed using Sidak’s multiple comparisons test. A *P* value of less than 0.05 was considered statistically significant. Significance levels are indicated as follows: *P ≤ 0.05, **P ≤ 0.01, ***P ≤ 0.001, ^#^*P* ≤ 0.05, ^##^*P* ≤ 0.01, and ^###^*P* ≤ 0.001.

## Results

### The long-term implantation of dTEVGs in humans results in inadequate regeneration, AIH, and PGF

We treated a patient in the late stage of chronic kidney disease who had previously undergone arteriovenous fistula creation using a dTEVG prepared from an allogeneic iliac artery. Due to the surgery not being performed at our institution, the specific details of the fistula procedure were unclear. Following a kidney transplant, the patient required fistula closure. After obtaining ethical approval and informed consent, we retrieved the dTEVG, which had been in use for 5 years. Native iliac artery specimens were collected from patients undergoing amputation procedures. The decellularized iliac artery was prepared by removing the cellular components from the native tissue. All tissue collection was approved by the ethics committee. Histological staining (HE, EVG, and Masson staining) was performed on dTEVGs and their original material, human iliac arteries. The dTEVGs retained the major extracellular matrix (ECM) components and maintained their structural integrity (Fig. S1).

**Fig. 1.**
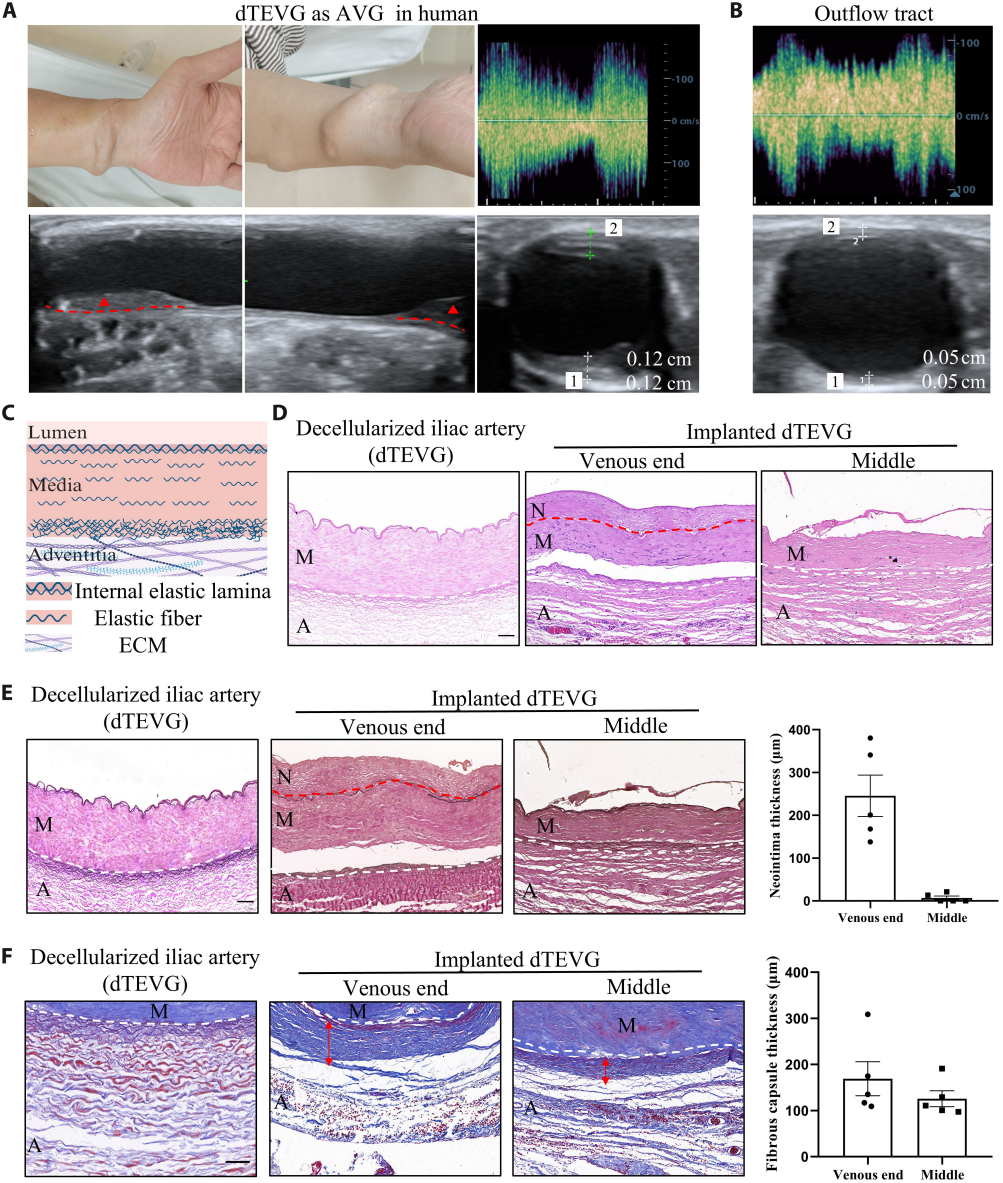
Decellularized tissue-engineered vascular grafts (dTEVGs) implanted in humans as arteriovenous grafts (AVGs) after 5 years resulted in inadequate regeneration, anastomotic intimal hyperplasia (AIH), and perigraft fibrosis (PGF). (A) The appearance and vascular ultrasound of the dTEVG after implantation in a human. Intimal hyperplasia is primarily observed at the anastomosis of the dTEVG, as indicated by the ultrasound. The red dashed line indicates the boundary between the intima and the media, and the red triangle highlights the area of AIH. (B) The vascular ultrasound of the venous outflow tract of the AVG (dTEVG). The degree of intimal hyperplasia is milder compared to AIH. (C) Schematic diagram of the tissue structure of the dTEVG. The dTEVG contains elastic fiber layers and an internal elastic lamina, both composed of elastic fibers. (D) Hematoxylin and eosin (HE) staining of the dTEVG before implantation and 5 years after implantation. Cell infiltration is observed in the dTEVG after 5 years, although it is not complete, and intimal hyperplasia is also observed. Scale bar, 100 μm. (E) Elastin–van Gieson (EVG) staining of the dTEVG before implantation and 5 years after implantation. Five years after implantation, the internal elastic lamina remains intact, the outer elastic fiber shows partial degradation, and significant AIH is observed at the venous end. Scale bar, 100 μm. (F) Masson staining of the dTEVG before implantation and 5 years after implantation. An obvious dense fibrous capsule is observed around the periphery of the dTEVG after implantation. Scale bar, 100 μm. The red dashed line indicates the boundary between the neointima and the media, while the white dashed line indicates the boundary between the media and the adventitia. N represents the neointima, M represents the media, and A represents the adventitia. ECM, extracellular matrix.

When used as an AVG, the dTEVG was connected between the artery and vein at the wrist (Fig. 1A). The ultrasound spectrum demonstrated the patency of the dTEVG after 5 years (Fig. 1A and B). Additionally, the ultrasound images revealed that intimal hyperplasia (IH) was primarily localized at the anastomosis site of the dTEVG compared to the venous outflow tract connected to the dTEVG.

Similar to other dTEVGs prepared using large animal vessels [7], the dTEVG prepared from human iliac arteries exhibited dense elastic fiber layers, including an internal elastic lamina on the luminal side (Fig. 1C and Fig. S1). After 5 years of implantation, the elastic fiber layers remained largely intact, except for less degradation of the elastic fibers near the adventitia (Fig. 1D and E). Histological analyses were performed to further evaluate the cellular and extracellular changes within the dTEVG. Cellular infiltration was observed inside the dTEVG, although it was not complete (Fig. 1D). The dTEVG exhibited significant AIH (within the red dashed line) (Fig. 1E). Additionally, a distinct PGF was observed (Fig. 1F).

In summary, the long-term in vivo performance of dTEVGs that had been implanted as AVGs in humans for 5 years revealed several key pathological features, including inadequate regeneration, AIH, and PGF. These factors may contribute to the long-term functional limitations of the graft when used as an AVG.

### AIH and PGF in the dTEVG-AVG rat model

The lack of suitable small animal models is a significant challenge in studying the in vivo remodeling process of dTEVGs. Due to ethical limitations, we are often unable to obtain patent, functioning AVG samples from patients. Therefore, this study employed the novel rat dTEVG-AVG model for further in vivo investigation. Our previous studies have demonstrated that the rat dTEVG-AVG model is an appropriate small animal model for simulating clinical phenotypes and exhibits pathological phenomena similar to those in humans [15]. In the rat model, we selected samples collected on day 21 postimplantation, as this time point had passed the postimplantation acute stage and exhibited phenotypes similar to those observed in human samples.

**Fig. 2.**
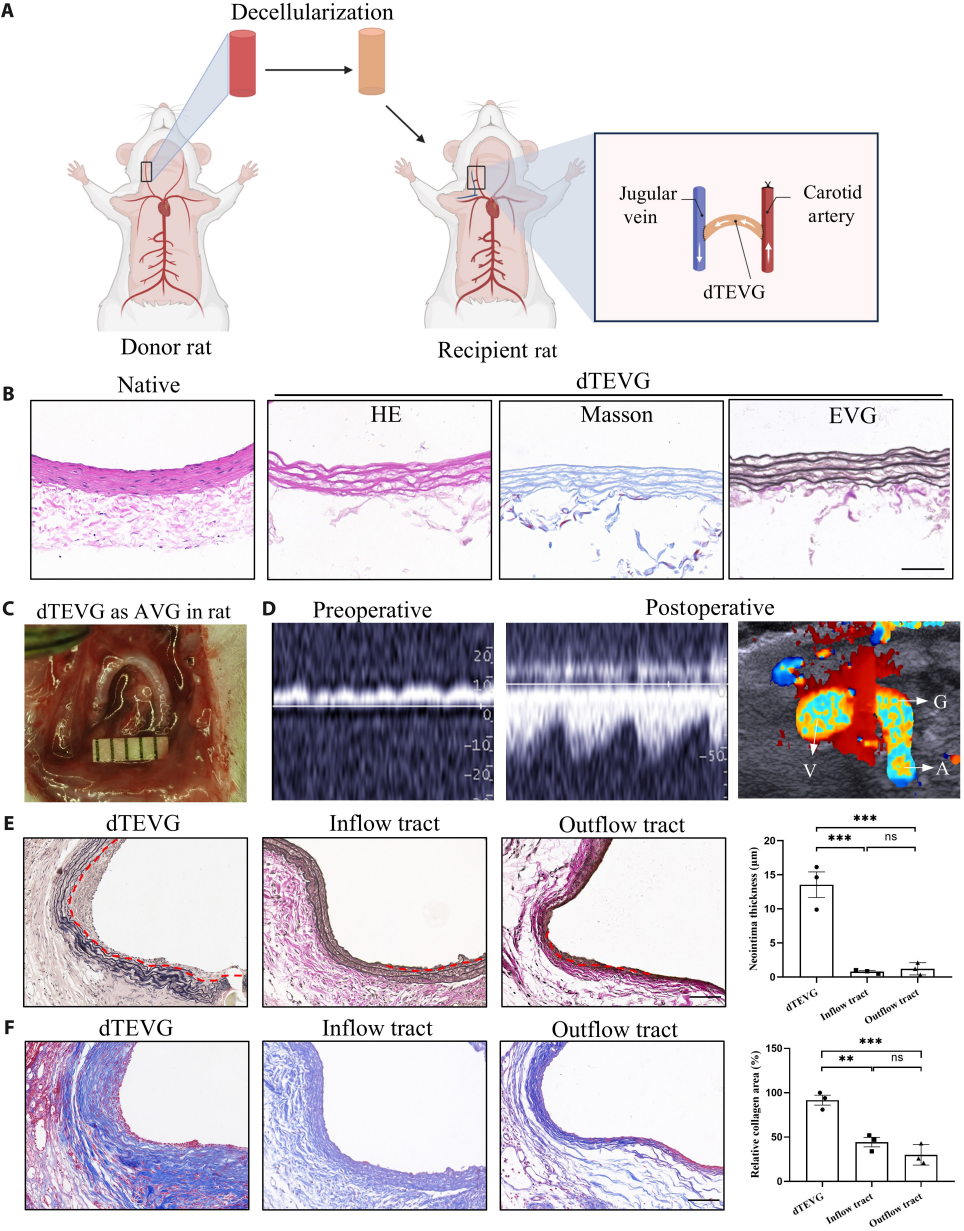
Establishment of the dTEVG-AVG rat model and characterization of the AIH and PGF of dTEVGs. (A) Schematic illustration of the establishment of the dTEVG-AVG rat model. (B) Histological staining of rat carotid arteries before and after decellularization. Scale bar, 50 μm. (C) dTEVG 21 d postsurgery in the dTEVG-AVG rat model. (D) Noninvasive ultrasound imaging of the venous outflow before and after surgery shows the patency of the model, with color Doppler imaging indicating a semicircular shape of the dTEVG in vivo. (E) EVG staining of the dTEVG and inflow tract and outflow tract of the AVG in a rat. Significant intimal hyperplasia is observed at the anastomosis of the dTEVG rather than the inflow tract (carotid artery) and outflow tract (internal jugular vein) of the AVG. The red dashed line indicates the boundary between the neointima and the media. Scale bar, 100 μm. (F) Masson staining of the dTEVG and the inflow tract and outflow tract of the AVG in a rat. An obvious PGF of the dTEVG. Scale bar, 100 μm.

A schematic illustration of the establishment of the dTEVG-AVG rat model is shown in Fig. 2A. Decellularized rat CAs were used as dTEVGs. Histological staining of the CAs before and after decellularization showed complete removal of cellular structures while preserving elastic and collagen fibers (Fig. 2B). The macroscopic appearance of the grafts in the dTEVG-AVG rat model 21 d postsurgery is shown in Fig. 2C. The dTEVG in the rat model remained patent, as confirmed by noninvasive ultrasound imaging, which showed the dTEVG maintaining a semicircular shape in vivo (Fig. 2D).

It is noteworthy that the dTEVG exhibited pathological phenomena similar to those observed in human samples. EVG staining revealed significant AIH of the dTEVG, while no such changes were observed in the inflow tract (CA) or outflow tract (internal jugular vein) of the AVG (Fig. 2E). Inadequate regeneration was also observed (Fig. 2E). Additionally, Masson staining highlighted prominent PGF of the dTEVG (Fig. 2F). These findings demonstrate the successful establishment of the dTEVG-AVG rat model, along with notable pathological features such as AIH and PGF.

### M2 macrophage infiltration in the AIH and PGF area in the dTEVG-AVG of humans and rats

**Fig. 3.**
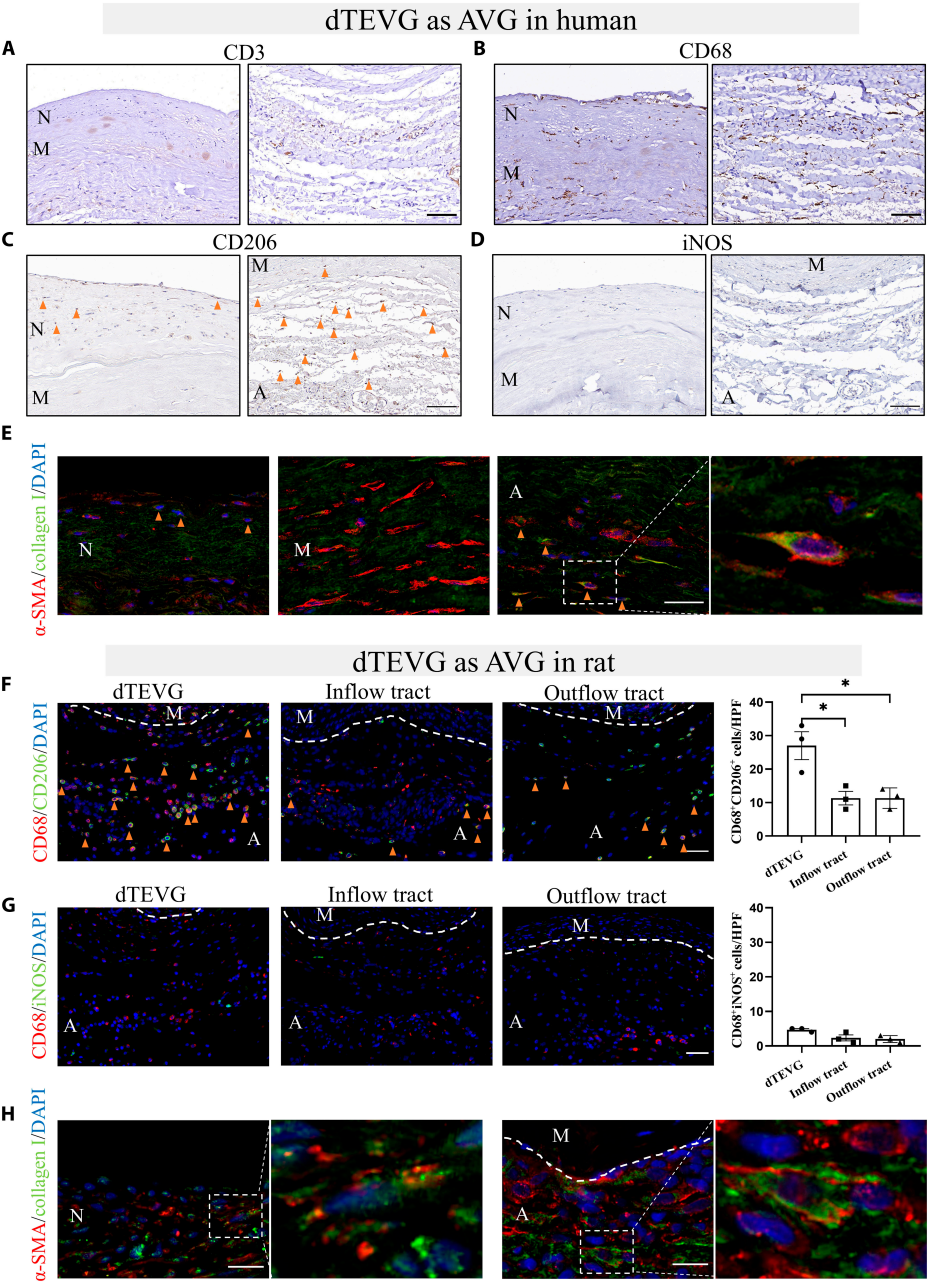
M2 macrophage and myofibroblast infiltration in dTEVGs as AVGs in humans and rats at late stages. (A) The immunohistochemistry (IHC) of CD3 characterizes T cell infiltration in the dTEVG 5 years after implantation in humans. Scale bar, 100 μm. (B) The IHC of CD68 characterizes macrophage infiltration in the dTEVG 5 years after implantation in humans. Compared to T cells, a higher infiltration of macrophages is observed. Scale bar, 100 μm. (C) The IHC of CD206 characterizes M2 macrophage infiltration in the dTEVG in humans. Scale bar, 100 μm. (D) The IHC of iNOS characterizes M1 macrophage infiltration in the dTEVG in humans. There is less infiltration of M1 macrophages in the dTEVG. Scale bar, 100 μm. (E) Immunofluorescence (IF) characterizes myofibroblast infiltration in the intima, media, and adventitia of the dTEVG. Infiltration of myofibroblasts (α-SMA and collagen I double-positive cells) is observed in the neointima and adventitia of the dTEVG. Scale bar, 50 μm. (F) IF characterizes M2 macrophage infiltration in the dTEVG and the inflow tract and outflow tract of the AVG in rats after 21-d implantation. A significantly higher infiltration of M2 macrophages is observed at the periphery of the dTEVG. Scale bar, 50 μm. (G) IF characterizes M1 macrophage infiltration in the dTEVG and the inflow tract and outflow tract of the AVG in rats after 21-d implantation. Infiltration of M1 macrophages is not evident at 21 d postimplantation, and no differences are observed between the 3 different regions. Scale bar, 50 μm. (H) IF characterizes the myofibroblast infiltration in the intima, media, and adventitia of the dTEVG in rats. The media of the dTEVG shows a lack of cellular infiltration. The infiltration of myofibroblasts is observed in the neointima and adventitia. Scale bar, 50 μm. The white dashed line indicates the boundary between the media and the adventitia. N represents the neointima, M represents the media, and A represents the adventitia. The orange triangle indicates positive cells. DAPI, 4′,6-diamidino-2-phenylindole. HPF, high power field.

We aimed to find the causes of the observed pathological phenomena in dTEVGs. Given the important regulatory role of immune cells in graft remodeling, particularly macrophages and T cells, we first performed immunohistochemistry to assess macrophage and T cell infiltration in dTEVG samples postimplantation in humans (Fig. 3A and B). We found that CD68-positive macrophages infiltrating the dTEVG were significantly higher in number than CD3-positive T cells, and macrophage infiltration was also present in the neointima (Fig. 3A and B). We then further assessed the polarization of the infiltrating macrophages and found that the majority were CD206-positive M2 macrophages (Fig. 3C and D). We performed the same characterization in the rat dTEVG-AVG model. IF revealed significant infiltration of M2 macrophages in the dTEVG in rats, compared to that in the inflow tract (CA) and outflow tract (jugular vein) connected to the dTEVG (Fig. 3F). No significant difference in M1 macrophage infiltration was observed among the 3 sites (Fig. 3G).

Given the presence of AIH and PGF observed in the dTEVG, together with the role of myofibroblasts as key effector cells in other IH- or fibrosis-related diseases, we then evaluated the presence of myofibroblasts in the dTEVG. As we predicted, we identified myofibroblasts (α-SMA- and collagen-positive cells) in both the neointima and adventitia of the dTEVG removed from our patient (Fig. 3E). We then assessed the infiltration of myofibroblasts in the rat model and found mutually corroborative evidence that the infiltration in both the neointima and adventitia was similar to that in the dTEVG in humans (Fig. 3H). This suggests that tissue regeneration and remodeling is occurring at the dTEVG.

### Construction of a rat model for continuous promotion of M2 macrophage infiltration in dTEVG-AVG

We found that the dTEVG with dense elastic fiber layers exhibited significant infiltration of M2 macrophages during the remodeling process when used as dialysis access (Fig. 3). Furthermore, the regeneration within the dTEVG was incomplete (Figs. 1 and 2). Since previous studies have shown that M2 macrophages play a role in promoting graft remodeling [13,18], we hope to investigate whether enhancing M2 macrophage infiltration can improve regeneration in the dTEVG.

To establish a stable and long-term sustained-release environment in vivo for the continuous promotion of M2 macrophage polarization, we loaded the classic M2 macrophage-polarizing cytokine IL-4 into PLGA NPs (IL-4 NP) and combined them with GelMA hydrogel to construct a sustained-release system (Fig. S2). A schematic illustration of the preparation of IL-4 NP-GelMA hydrogel is shown in Fig. S2A and B. First, we prepared PLGA NPs using the nanoprecipitation method and obtained their powdered form through lyophilization. Next, we incorporated PLGA NPs into the GelMA hydrogel precursor solution and prepared the IL-4 NP-GelMA hydrogel system after photopolymerization. We characterized the morphology of PLGA NPs and IL4@PLGA NPs using transmission electron microscopy (Fig. S2C). SEM was employed to evaluate the microstructure of the unloaded GelMA hydrogel and IL-4 NP-GelMA hydrogel system (Fig. S2D). PLGA NPs were deposited on the inner surface of the hydrogel, and their incorporation did not disrupt the fundamental structure of the gel. The diameter of unloaded PLGA NPs was 175.10 ± 65.42 nm, while the diameter of IL-4@PLGA NPs increased to 277.70 ± 93.57 nm (Fig. S2E). The drug loading capacity of the NPs was approximately 3.98% ± 0.02%, with an encapsulation efficiency of 62.47% ± 0.14%. Finally, we assessed the drug release performance of this system. As shown in Fig. S2G, the system did not exhibit a “burst-release” phenomenon, achieving continuous release of IL-4. By day 21, approximately 80% of the drug had been released, demonstrating the long-term stable release capability of the IL-4 NP-GelMA hydrogel system.

**Fig. 4.**
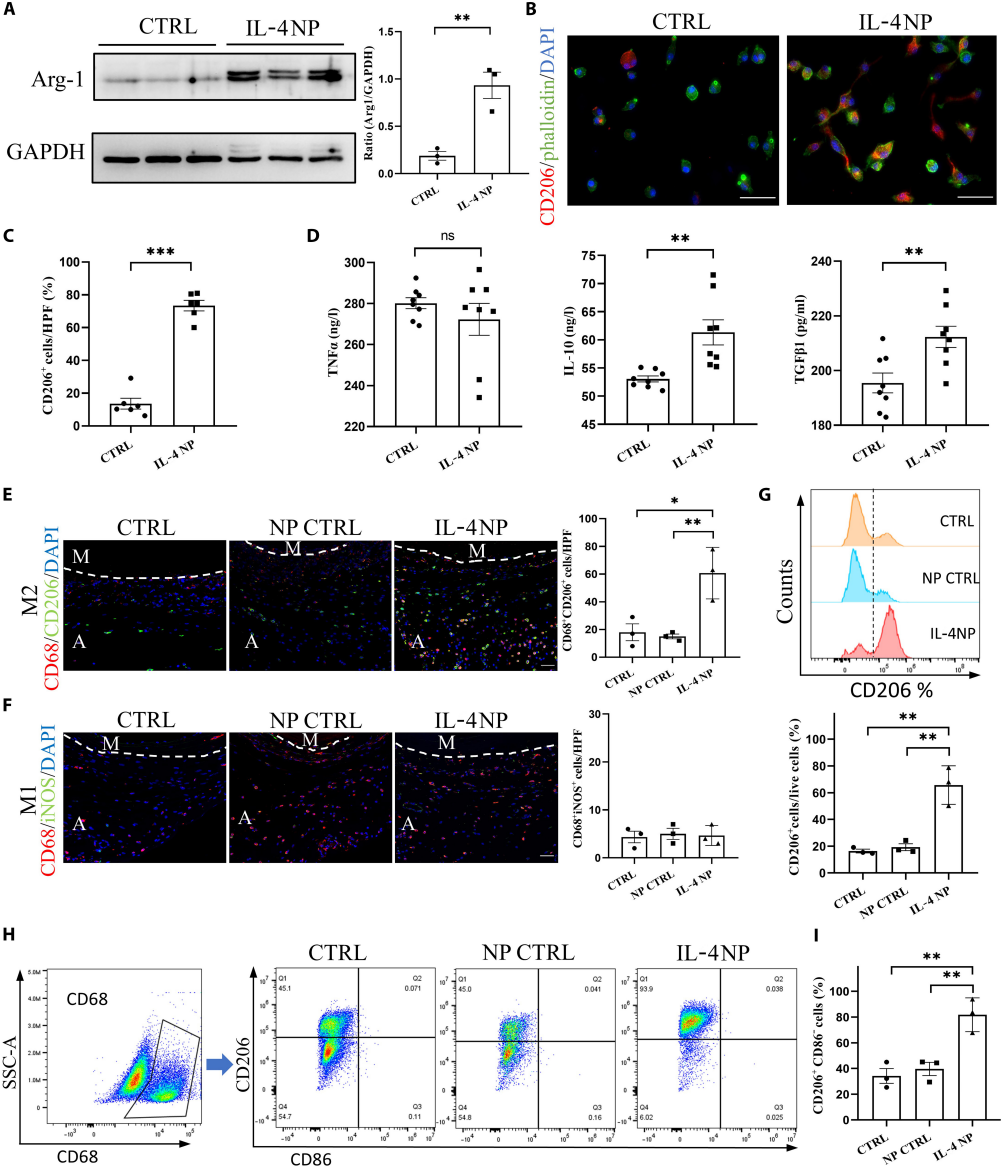
Long-term capability of promoting M2 macrophage polarization of IL-4 nanoparticles (NPs) in vitro and in vivo. (A) Increased Arg-1 expression in bone-marrow-derived macrophages (BMDMs) after intervention with the culture medium extracted from IL-4 NP-GelMA hydrogel on day 14. CTRL represents the unloaded GelMA hydrogel (*n* = 3). (B) The number of CD206-positive cells increases, and the cells exhibit a more elongated morphology in BMDMs after intervention with the culture medium extracted from IL-4 NP-GelMA hydrogel on day 14. Scale bar, 50 μm. (C) Statistical results of CD206^+^ cells (*n* = 6). (D) Enzyme-linked immunosorbent assay (ELISA) results showing a significant increase in IL-10 and TGF-β1 expression in the cell supernatant after IL-4 NP-GelMA hydrogel intervention (*n* = 8). (E) IF characterizes the infiltration of M2 macrophages at the periphery of the dTEVG 21 d after IL-4 NP-GelMA hydrogel implantation (*n* = 3). The CTRL group underwent surgery without any intervention. The NP CTRL group received PLGA NP-GelMA hydrogel without IL-4. The IL-4 NP group, which received IL4 NP-GelMA hydrogel, showed significant infiltration of M2 macrophages. Scale bar, 50 μm. (F) IF characterizes the infiltration of M1 macrophages at the periphery of the dTEVG 21 d after IL-4 NP-GelMA hydrogel implantation (*n* = 3). Infiltration of M1 macrophages showed no significant differences among the 3 groups. Scale bar, 50 μm. The white dashed line indicates the boundary between the media and the adventitia. M represents the media, and A represents the adventitia. (G) Flow cytometry of in vivo samples showing a significant increase in the proportion of CD206^+^ cells after IL-4 NP-GelMA hydrogel intervention. (H) CD206 and CD86 expression in CD68^+^ cell populations. (I) Quantification showing a significant increase in the proportion of CD206^+^CD86^−^ cells after IL-4 NP-GelMA hydrogel intervention (*n* = 3).

We first verified the ability to promote M2 macrophage polarization of the system in vitro using rat BMDMs. The IL-4 NP-GelMA hydrogel system was soaked in cell culture medium, with the medium replaced every 24 h until day 14. On day 14, the system was placed in fresh culture medium for 24 h, and the soaked medium was then used to treat BMDMs, while the control group used ordinary cell culture medium (Fig. S2H). We observed a significant increase in the expression of Arg-1 (a classic M2 macrophage marker) in the IL-4 NP group (Fig. 4A). IF of CD206 (M2 macrophage marker) and phalloidin indicated that the number of CD206^+^ cells was markedly higher, and cells presented M2 macrophage morphology after treatment (Fig. 4B and C). Additionally, ELISA analysis of the cell supernatant revealed a significant increase in IL-10 (an anti-inflammatory molecule primarily secreted by M2 macrophages) after treatment. TNFα (a pro-inflammatory factor primarily secreted by M1 macrophages) showed a slight decrease, although this result was not statistically significant. Furthermore, TGF-β1 secretion (primarily secreted by M2 macrophages) was also significantly elevated after treatment (Fig. 4D). Overall, these results indicate that IL-4 NP-GelMA hydrogel can effectively promote long-term M2 macrophage polarization in vitro.

We applied the IL-4 NP-GelMA hydrogel to the rat dTEVG-AVG model on day 0. Specifically, the system was placed near the dTEVG area in the rats’ neck region to ensure that the released IL-4 could penetrate the dTEVG area without affecting the migration of surrounding tissue cells to the dTEVG. The CTRL group underwent surgery without any intervention. The NP CTRL group received PLGA NP-GelMA hydrogel without IL-4.

We observed that compared to the CTRL group and NP CTRL group, the IL-4 NP group significantly promoted M2 macrophage infiltration around the dTEVG area on day 21 (Fig. 4E), with no significant effect on M1 polarization (Fig. 4F). Moreover, after IL-4 NP intervention, there was no significant change in the infiltration of CD3-positive T cells at the periphery of the dTEVG, indicating that the IL-4 NP-GelMA hydrogel primarily regulates M2 macrophage infiltration (Fig. S3A).

After IL-4 NP delivery, the proportion of CD206^+^ cells within the total cell population was significantly increased (Fig. 4G), while the proportion of CD86^+^ cells remained low and showed no significant difference between groups (Fig. S4B). Further analysis gated on CD68^+^ cells (macrophage lineage) showed a significant increase in CD206^+^CD86^−^ macrophages in the IL-4 NP-treated group (Fig. 4H and I). In addition, we examined the proportions of CD68^+^CD206^+^ and CD68^+^CD86^+^ subpopulations (Fig. S4C and D). The CD68^+^CD206^+^ population increased significantly following IL-4 NP intervention, whereas the CD68^+^CD86^+^ population remained low and unchanged between groups. These results demonstrate that the IL-4 NP-GelMA hydrogel has the capability to long-term regulate macrophage polarization toward M2 macrophages in the rat dTEVG-AVG model in vivo.

### Enhancing M2 macrophage infiltration promotes AIH and PGF

**Fig. 5.**
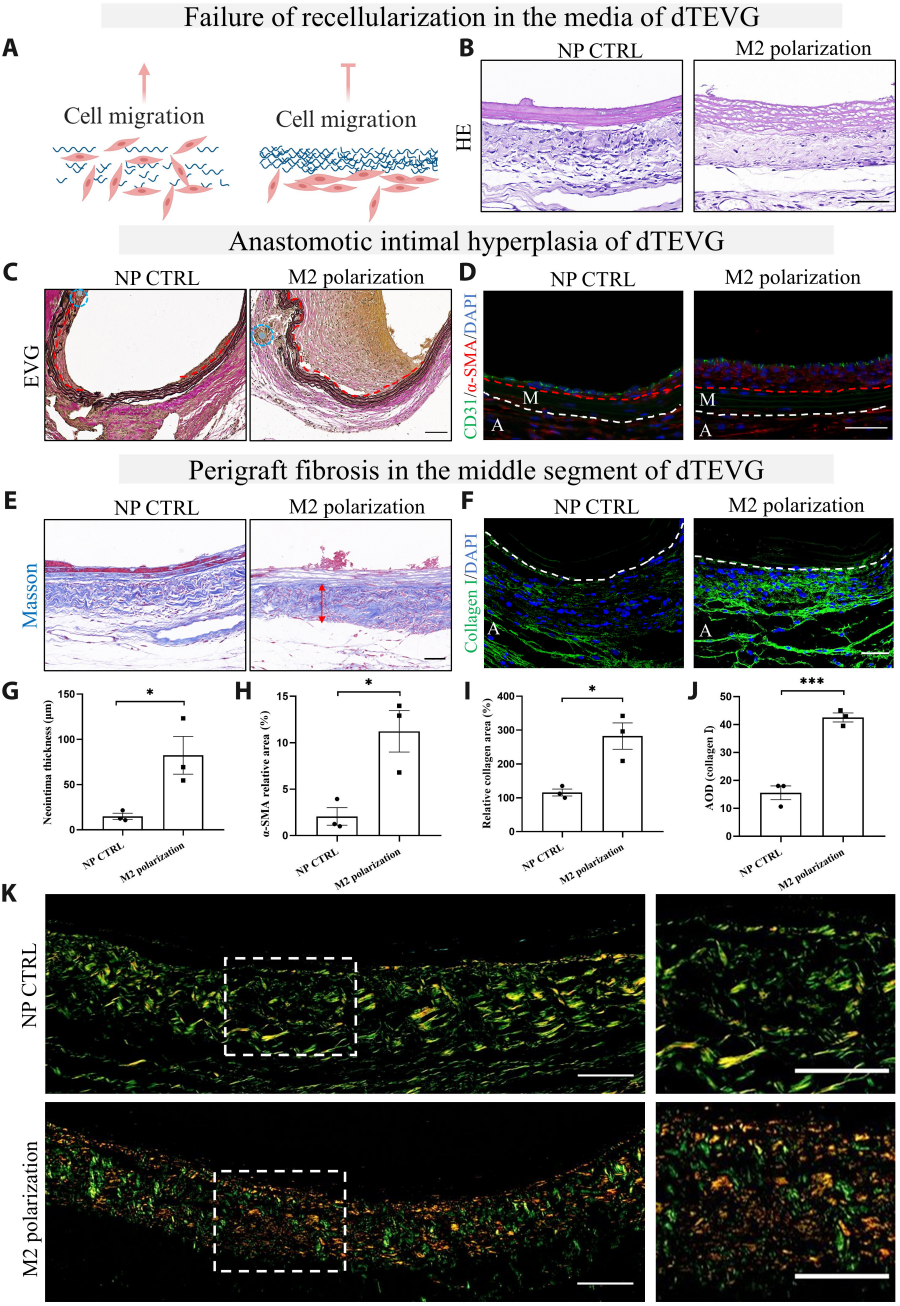
M2 macrophage infiltration promotes AIH and PGF in the rat dTEVG-AVG model. (A) Schematic diagram of the hypothesis that dense elastic fiber layers hinder cell migration. (B) HE staining of the middle segment of the dTEVG before and after promoting M2 macrophage infiltration. Enhanced infiltration of M2 macrophages did not increase cellular infiltration within the dTEVG (media). Scale bar, 50 μm. (C) EVG staining of the anastomosis site of the dTEVG. The AIH of the dTEVG was more severe after enhancing M2 macrophage infiltration. The blue circle indicates the suture section at the anastomosis. Scale bar, 50 μm. (D) IF of CD31 and α-SMA at the anastomosis site of the dTEVG. There was an increased neointima after enhancing M2 macrophage infiltration. Scale bar, 50 μm. (E) Masson staining of the middle segment of the dTEVG. The PGF of the dTEVG was further increased after enhancing M2 macrophage infiltration, along with fibrous encapsulation. Scale bar, 50 μm. (F) IF of collagen I of the middle segment of the dTEVG. Expression of collagen I increased after enhancing M2 macrophage infiltration. Scale bar, 50 μm. (G to J) The statistical analysis results of panel (C) (G), panel (D) (H), panel (E) (I), and panel (F) (J). (K) Sirius Red staining of the middle segment of the dTEVG. The deposition of collagen I (in red) increased after enhancing M2 macrophage infiltration. Scale bar, 50 μm. The red dashed line indicates the boundary between the neointima and the media, while the white dashed line indicates the boundary between the media and the adventitia. M represents the media, and A represents the adventitia. AOD, average optical density.

We found that the dTEVG exhibited incomplete regeneration within its structure (Figs. 1 and 2). The dense fibrous structure of the graft has been shown to hinder cell infiltration [19]. Conversely, moderate degradation of the elastic fiber layer has been demonstrated to promote regeneration [20]. Therefore, we hypothesize that the incomplete infiltration is due to the dense elastic fiber layers in the dTEVG, which block cell infiltration (Fig. 5A). Previous studies highlighted the role of M2 macrophages in promoting tissue regeneration [13,18]. Because of the poor cell infiltration within the dTEVG, we aim to promote the regeneration of the dTEVG by enhancing M2 macrophage infiltration. Surprisingly, there was no increase in cell infiltration within the graft (the media) after enhancing M2 macrophage infiltration (Fig. 5A and B).

Next, we aim to investigate whether enhanced M2 macrophage infiltration has other effects. We evaluated the anastomosis site and middle segment of the dTEVG. After promoting M2 macrophage infiltration, the neointima at the anastomosis site significantly increased (Fig. 5C). IF of CD31 and α-SMA confirmed the presence of the neointima (Fig. 5D). Furthermore, the PGF of the dTEVG middle segment increased, and a fibrous capsule formed (Fig. 5E). IF staining for collagen I confirmed the increased collagen deposition (Fig. 5F). Mature and immature collagen exhibited different colors under polarized light after Sirius Red staining. Collagen type I appeared orange-red or red, while collagen type III appeared green. We found that after promoting M2 macrophage infiltration, the deposition of collagen I the dTEVG periphery increased, indicating that it was in a state of PGF (Fig. 5K).

In summary, we found that promoting M2 macrophage infiltration did not result in recellularization of the dTEVG but instead contributed to AIH and increased PGF. Next, we conducted further studies to investigate the underlying causes of AIH and PGF.

### Enhanced M2 macrophage infiltration leads to myofibroblast activation in dTEVGs

Myofibroblasts are considered the primary effector cells in fibrosis-related disease [21–23] and have been identified as one of the cell sources of the neointima in coronary artery injury models [24]. Furthermore, myofibroblast activation affects organ structural and functional changes through its fibrotic capabilities [23,25–27]. A few studies have found that myofibroblast activation is accompanied by increased expression of the M2 macrophage marker CD206 [21,28]. In both our human samples and mouse models, the common infiltration of M2 macrophages and myofibroblasts at the IH and PGF area was demonstrated. Therefore, we hypothesized that enhancing M2 macrophage infiltration may promote myofibroblast activation, which leads to PGF. Additionally, due to the barrier effect of the dense elastic fiber layers, activated myofibroblasts migrated to the anastomosis site, moving through gaps toward the lumen, thereby contributing to AIH.

**Fig. 6.**
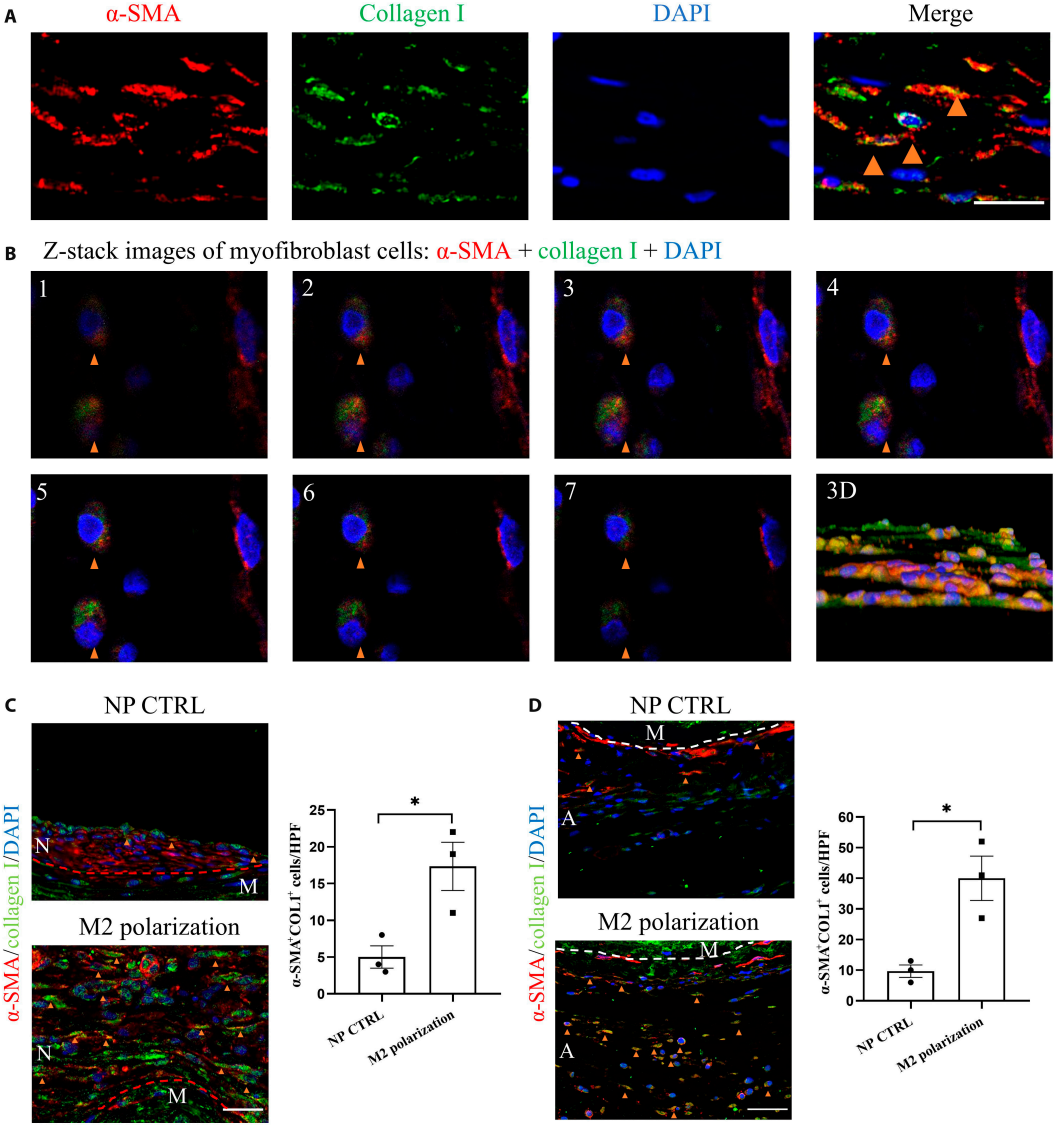
Enhanced M2 macrophage infiltration leads to myofibroblast activation in dTEVGs. (A) IF of α-SMA (red) and collagen I (green) in the adventitia of the dTEVG. Scale bar, 20 μm. (B) Z-stack images of 3-color confocal microscopy identifies cells coexpressing α-SMA (red) and collagen I (green), indicating that these α-SMA-positive cells have the ability to produce type I collagen, which is a hallmark of myofibroblasts. Panels 1 to 7 show different layers of fluorescent confocal results, and the 3-dimensional (3D) panel shows the 3D reconstruction of the cells. Yellow arrows indicate positive cells (myofibroblasts). (C) Myofibroblast infiltration in the neointima increased after promoting M2 macrophage infiltration. Scale bar, 30 μm. (D) Myofibroblast infiltration in the adventitia of the dTEVG increased after promoting M2 macrophage infiltration. Scale bar, 30 μm. The red dashed line indicates the boundary between the neointima and the media, while the white dashed line indicates the boundary between the media and the adventitia. N represents the neointima, M represents the media, and A represents the adventitia.

We first assessed the infiltration of myofibroblasts before and after promoting M2 macrophage infiltration. Myofibroblasts are typically characterized by the expression of α-SMA and collagen I [22]. Three-color confocal microscopy identified a cluster of cells in the dTEVG that coexpressed α-SMA and collagen I, indicating that these α-SMA-positive cells have the ability to produce type I collagen, which is a hallmark of myofibroblasts (Fig. 6A). We further analyzed the positive cells with Z-stack analysis and confirmed the coexpression (Fig. 6B). Panels 1 to 7 show different layers of fluorescent confocal results, and the 3-dimensional (3D) panel shows the 3D reconstruction of the cells. Furthermore, we found that after promoting M2 macrophage infiltration, the number of myofibroblasts in both the neointima and adventitia of the dTEVG significantly increased (Fig. 6C and D).

The above results indicate that enhanced M2 macrophage infiltration leads to myofibroblast activation. As a result, it contributes to IH and PF, which are considered adverse outcomes for the long-term patency of dTEVGs. Here, we revealed the dual role of M2 macrophages: they facilitate AVG remodeling but also bring about adverse outcomes due to the overactivation of myofibroblasts. Next, we aim to identify the specific signaling pathways through which M2 macrophage infiltration activates myofibroblasts, thus minimizing the potential risk of AIH and PGF in vivo.

### M2 macrophage infiltration promotes myofibroblast activation via the TGF-β1/Smad3 signaling pathway

M2 macrophages are considered to participate in tissue repair and regeneration by producing cytokines such as TGF-β1 (Fig. 4D) [29]. In renal fibrosis, studies have found that the TGF-β1/Smad3 pathway is a key signaling route for the activation of myofibroblasts [21,28]. We hypothesize that the TGF-β1/Smad3 signaling pathway is a crucial mechanism through which M2 macrophages activate myofibroblasts in dTEVGs.

**Fig. 7.**
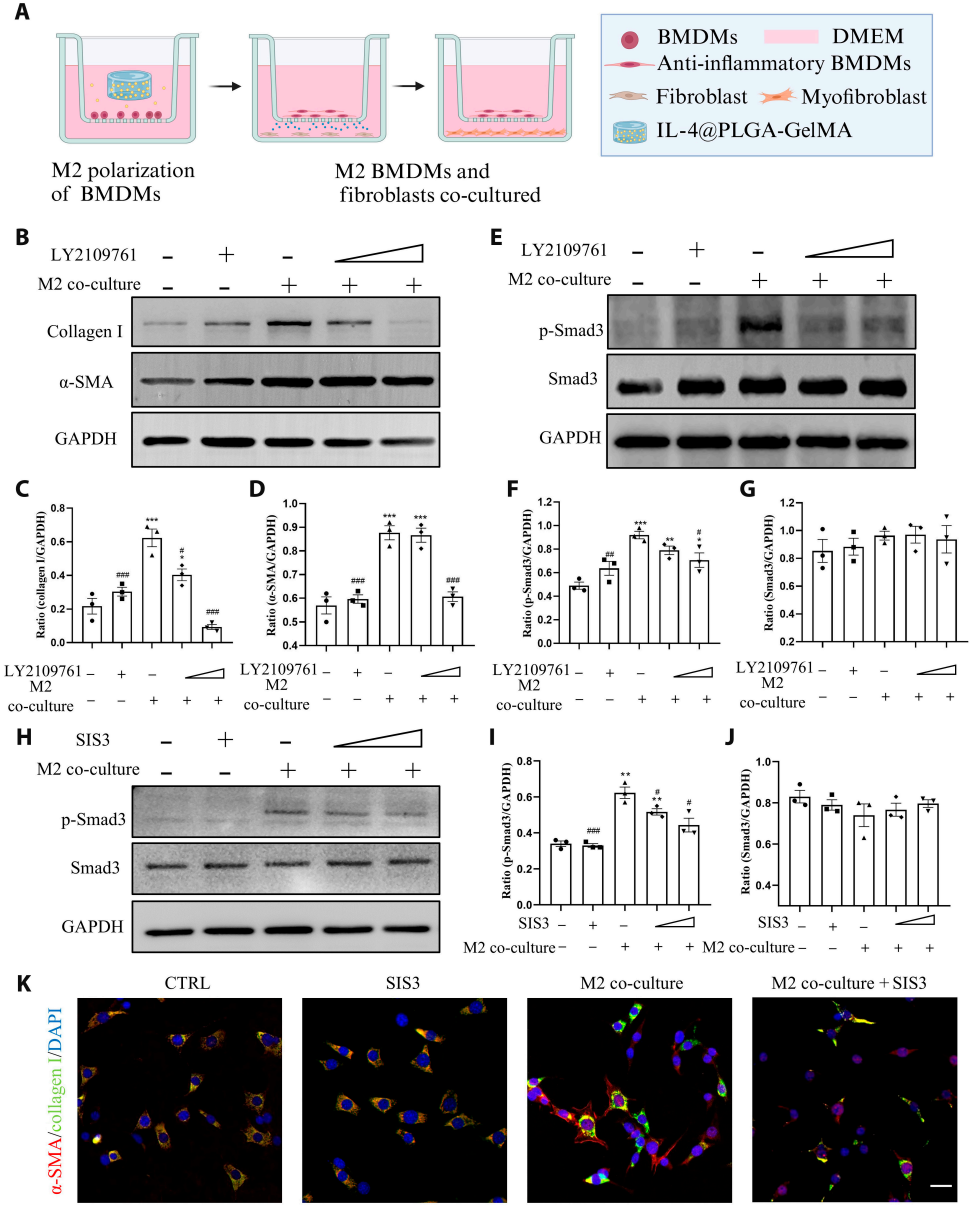
In vitro co-culturing of M2 macrophages with fibroblasts reveals that M2 macrophages promote myofibroblast activation via the TGF-β1/Smad3 signaling pathway. (A) Schematic of the in vitro co-culture experiment with M2 macrophages and fibroblasts. (B) M2 macrophage co-culture enhances the expression of collagen I and α-SMA proteins in NIH 3T3 fibroblast cells, while the TGFβR1 inhibitor LY2109761 (1 and 10 μM) inhibits this effect. (C and D) Relative quantification of collagen I (C) and α-SMA (D) protein levels in NIH 3T3 cells after intervention. (E) M2 macrophage co-culture enhances the phosphorylation of Smad3 in NIH 3T3 fibroblast cells, while the TGFβR1 inhibitor LY2109761 (1 and 10 μM) inhibits this effect. This suggests activation of the TGF-β1/Smad3 signaling pathway. (F and G) Relative quantification of p-Smad3 (F) and Smad3 (G) protein levels in NIH 3T3 cells after intervention. (H) M2 macrophage co-culture enhances the phosphorylation of Smad3 in NIH 3T3 fibroblast cells, while the Smad3 phosphorylation inhibitor SIS3 (1 and 10 μM) inhibits this effect. (I and J) Relative quantification of p-Smad3 (I) and Smad3 (J) protein levels in NIH 3T3 cells after intervention. (K) IF characterized cell morphology and coexpression of α-SMA and collagen I. NIH 3T3 cells were treated with SIS3 (10 μM), M2 macrophage co-culture, or M2 macrophage co-culture + SIS3 (10 μM), while the control group used ordinary cell culture medium. Scale bar, 50 μm. * indicates comparison with the “no M2 macrophage co-culture, no LY2109761 group”, and # indicates comparison with the “only M2 macrophage co-culture group”. DMEM, Dulbecco’s modified Eagle’s medium.

We simulated the in vivo enhanced M2 macrophage infiltration microenvironment by co-culturing fibroblasts with M2 macrophages in vitro (Fig. 7A). Firstly, we used the IL-4 NP-GelMA hydrogel in a transwell chamber to polarize BMDMs into M2 macrophages. Then, we co-cultured the M2 macrophages with fibroblasts. We observed that co-culturing with M2 macrophages significantly increased the expression of collagen I and α-SMA in NIH 3T3 fibroblasts, while the TGFβR1 inhibitor LY2109761 inhibits this effect (Fig. 7B). Additionally, co-culturing with M2 macrophages resulted in an increase in Smad3 phosphorylation, a key event in the activation of the TGF-β1/Smad3 pathway (Fig. 7E). The use of the LY2109761 and Smad3-specific phosphorylation inhibitor SIS3 effectively inhibited the activation induced by co-culturing with M2 macrophages (Fig. 7E and H). IF showed that fibroblasts co-cultured with M2 macrophages exhibited a marked increase in α-SMA expression, and the cells displayed a more elongated morphology, resembling typical myofibroblasts. These changes were inhibited after suppressing the TGF-β1/Smad3 pathway (Fig. 7K). These results indicate that myofibroblast activation induced by M2 macrophage infiltration depends on the TGF-β1/Smad3 signaling pathway.

### Inhibition of Smad3 phosphorylation suppresses AIH and PGF without compromising M2 macrophage infiltration

We have discovered that M2 macrophage promotes myofibroblast activation through the TGF-β1/Smad3 signaling pathway. Next, we aim to investigate whether inhibiting the TGF-β1/Smad3 signaling pathway can suppress myofibroblast activation, as well as AIH and PGF at the anastomotic site, induced by enhanced M2 macrophage infiltration in the dTEVG. Smad3 phosphorylation is a key step in the activation of the TGF-β1/Smad3 signaling pathway [30,31]. To inhibit this pathway, we administered the specific Smad3 phosphorylation inhibitor SIS3 continuously via intraperitoneal injection for 21 d in the rat dTEVG-AVG model.

**Fig. 8.**
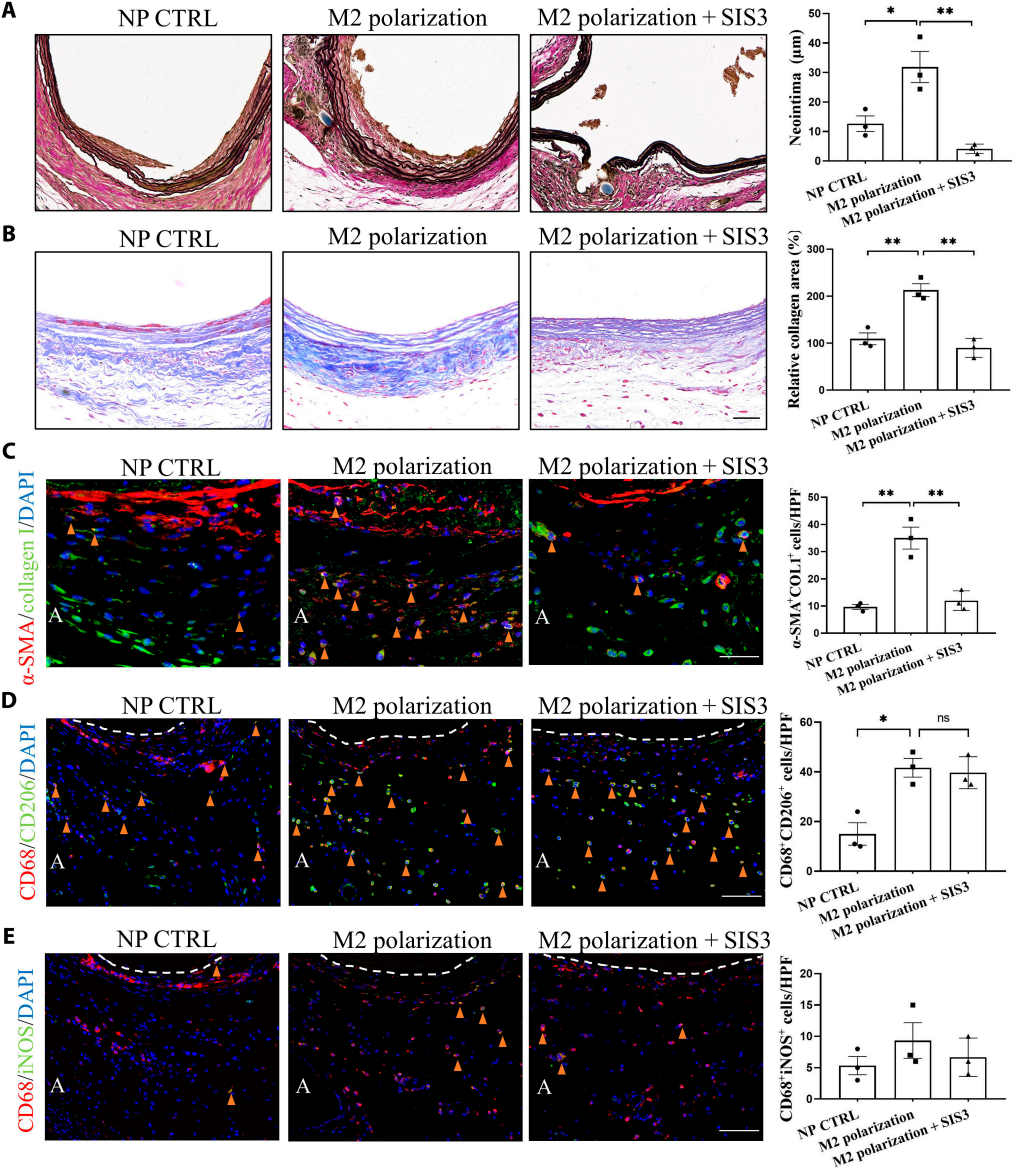
Inhibition of the Smad3 phosphorylation suppressed AIH and PGF caused by enhanced M2 macrophage infiltration in vivo, without affecting M2 macrophage infiltration. (A) EVG staining of dTEVGs in the NP CTRL, M2 macrophage polarization (IL-4 NP), and M2 macrophage polarization + SIS3 groups. SIS3 (2 mg/kg) was administered continuously via intraperitoneal injection for 21 d. The blue area represents the suture section at the anastomosis. Statistical analysis shows that the TGF-β1/Smad3 pathway inhibitor SIS3 inhibited the AIH caused by enhanced M2 macrophage infiltration. Scale bar, 50 μm. (B) Masson staining of dTEVGs in the NP CTRL, M2 macrophage polarization, and M2 macrophage polarization + SIS3 groups. Statistical analysis shows that SIS3 inhibited the increased PGF of dTEVG caused by enhanced M2 macrophage infiltration. Scale bar, 50 μm. (C) IF of α-SMA and collagen I revealed the myofibroblast infiltration of dTEVGs in the NP CTRL, M2 macrophage polarization, and M2 macrophage polarization + SIS3 groups. SIS3 inhibited the increased myofibroblast infiltration caused by enhanced M2 macrophage infiltration in vivo. Scale bar, 50 μm. (D) IF of CD68 and CD206 revealed that administration of SIS3 did not influence the infiltration of M2 macrophages in vivo. Scale bar, 50 μm. (E) IF of CD68 and iNOS revealed that administration of SIS3 did not influence the infiltration of M1 macrophages in vivo. Scale bar, 50 μm. The white dashed line indicates the boundary between the media and the adventitia. A represents the adventitia. The orange triangle indicates positive cells.

EVG staining revealed that SIS3 significantly reduced the AIH of the dTEVG (Fig. 8A). Masson staining demonstrated that SIS3 inhibited the PGF in the dTEVG adventitia caused by enhanced M2 macrophage infiltration (Fig. 8B). We assessed myofibroblast infiltration following SIS3 treatment using IF and found that SIS3 significantly inhibited the generation of myofibroblasts (Fig. 8C). To determine whether SIS3 treatment affected the promotion of M2 macrophage infiltration, we assessed the infiltration of M2 and M1 macrophages. We found that SIS3 treatment had no effect on the infiltration of M2 macrophages or M1 macrophages (Fig. 8D and E). In addition, we also assessed the function of M2 macrophage and no significant change was found (Fig. S5).

These results suggest that myofibroblast activation is a key contributor to the AIH and PGF of the dTEVG. Enhancing M2 macrophage infiltration activates myofibroblasts via the TGF-β1/Smad3 signaling pathway, which exacerbates AIH and PGF. The specific inhibitor of the TGF-β1/Smad3 signaling pathway, SIS3, can suppress these pathological processes without compromising M2 macrophage infiltration. This also suggests that targeting myofibroblast activation could be a potential strategy to inhibit AIH and PGF in dTEVGs.

## Discussion

dTEVGs exhibit superior long-term patency rates compared to traditional polymer materials, such as expanded polytetrafluoroethylene, when used as AVGs. Previous studies have shown that dTEVGs face major challenges in clinical use such as AIH and PGF [6–9]. However, due to ethical constraints and limitations of animal models, research addressing these challenges in dTEVGs remains limited and the in vivo remodeling mechanisms of dTEVGs remain poorly understood. Through integrated human and novel rat AVG models, this study investigates the mechanistic link between macrophage polarization and AIH and PGF in dTEVGs. We found that (a) M2 macrophage infiltration promotes AIH and PGF in dTEVGs with dense elastic fiber layers; (b) enhanced M2 macrophages promote myofibroblast activation via the TGF-β1/Smad3 signaling pathway, which is the key process of AIH and PGF; and (c) targeting Smad3 phosphorylation attenuates M2 macrophage infiltration-driven AIH and PGF without affecting M2 macrophage infiltration.

Macrophages are effector cells in immune rejection responses [12,14,18,21]. In both acute injury and the adaptation of biomaterials in vivo, there is a typical temporal process of macrophage polarization toward the M2 macrophage phenotype [13,32,33]. It is generally believed that promoting M2 macrophage polarization can aid in material repair and regeneration [13,34,35]. However, it remains unclear whether enhancing M2 macrophage infiltration during remodeling can promote the regeneration of the dTEVG. We used the well-established and stable PLGA NPs and GelMA hydrogel to construct a controlled-release system [36], which enabled stable and long-term modulation of M2 macrophage polarization both in vitro and in vivo. We found that enhancing M2 macrophage infiltration promotes AIH and PGF in the dTEVG (Fig. 6). This suggests that the regulatory role of macrophages in dTEVGs with dense fibrous structures differs from other materials such as polycaprolactone. Additionally, this reminds us that while many studies focus on promoting tissue regeneration and repair through M2 macrophage polarization [32,33,37], the potential drawbacks of overinfiltration of M2 macrophages need to be treated cautiously. Since both AIH and PGF are critical issues in vascular diseases [9,11,38], it is essential to consider not only its beneficial effects but also the potential side effects.

Myofibroblasts are considered a source of IH in the coronary injury model [24], and their activation is a key factor in many fibrotic diseases [21–23]. Activated myofibroblasts may have enhanced migratory abilities and secrete collagen and other ECM components [32,39]. Moreover, we identified the co-presence of M2 macrophages and myofibroblasts in both AIH and PGF areas of dTEVGs (Figs. 7 and 8). The dense fiber layer structure of dTEVGs, which can hinder cell infiltration, plays an influential role in compromising graft regeneration through the fibrous structure [13,20,36]. In vitro experiments have demonstrated that increasing the porosity of electrospun scaffolds can improve cell infiltration [40]. This may explain why M2 macrophage infiltration leads to AIH rather than graft regeneration. The incomplete regeneration is due to the dense elastic fiber layers in the dTEVG, which hinder M2 macrophage infiltration. Under this blockade, peripheral cells, especially myofibroblasts activated by M2 macrophages, produce PGF and continuously migrate toward the anastomotic site, leading to AIH. Further studies are needed to validate this hypothesis. However, it is difficult to achieve highly homogeneous porosity adjustments in dTEVG. Furthermore, due to the clinical requirements for vascular diameter [2], the original materials for dTEVGs are usually large blood vessels from allogeneic or xenogeneic sources [6,8,41,42]. This highlights the critical importance of exploring new therapeutic targets from a mechanistic perspective to address key issues such as AIH.

The process of M2 macrophages activating myofibroblasts is regulated by the TGF-β1/Smad3 signaling pathway (Fig. 8). TGF-β1, a classic cytokine released by M2 macrophages, has been confirmed as a key factor in activating myofibroblasts [43]. The phosphorylation of Smad3 and its formation of a complex with Smad4, followed by nuclear translocation and binding to the regulatory regions of target genes, represents a critical step in TGF-β1 activation of fibrosis-related gene expression [44]. Small-molecule inhibitors targeting Smad3 phosphorylation, such as SIS3, have been developed for various diseases, including renal fibrosis, bone loss, and tumor drug resistance [45–47]. The safety of targeting Smad3 phosphorylation has also been validated. Our research found that targeting Smad3 phosphorylation attenuates M2 macrophage infiltration-driven AIH and PGF without affecting M2 macrophage infiltration itself. This work introduces a novel strategy to address complications, such as AIH and PGF, arising from M2 macrophage infiltration in elastin-rich dTEVGs.

The findings of this study raise the possibility of incorporating Smad3-targeting strategies, such as SIS3 delivery, into the design of next-generation biomaterials. Recent studies have demonstrated the feasibility of integrating SIS3 into advanced biomaterial-based delivery systems. For example, Lian et al. [47] developed a self-carried nanodrug (SCND-SIS3) with excellent biocompatibility and immunomodulatory effects for targeted lung cancer therapy. Qiu et al. [48] incorporated SIS3 into a photodynamic immunomodulator targeting the ECM to fabricate FPC@S, which effectively released SIS3 to inhibit fibrosis and prevent breast cancer metastasis. Wang et al. [49] engineered an injectable drug-loaded hybrid hydrogel containing SIS3 that combines sonodynamic and chemodynamic therapy, showing potential in suppressing tumor progression. Future studies focusing on optimizing delivery methods and evaluating long-term efficacy in vivo will be critical for translating this strategy into clinical applications.

This study still has several limitations. Due to ethical constraints, it is not feasible to retrieve dTEVG samples that remain patent and functional in patients. Conversely, when the dTEVG becomes occluded, clinical management typically does not involve surgical removal, as this would subject the patient to an additional invasive procedure and unnecessary healthcare costs. These factors greatly limit the availability of explanted human samples, thereby precluding further analyses. In addition, the rat model collected samples only on day 21, which does not fully capture the complexity of long-term remodeling observed in humans.

In summary, this study highlights the dual role of M2 macrophages in elastin-rich dTEVGs, where their infiltration potentially contributes to regeneration but unexpectedly drives AIH and PGF. The activation of myofibroblasts regulated by the TGF-β1/Smad3 signaling pathway is the underlying mechanism. Critically, pharmacological inhibition of Smad3 phosphorylation selectively disrupts this pathological cascade, reducing AIH and PGF without affecting macrophage recruitment.

## Data Availability

Data will be made available on request.
