# Supplementary material for: Targeting Smad3 Phosphorylation Attenuates Anastomotic Intimal Hyperplasia and Perigraft Fibrosis in Decellularized Tissue-Engineered Vascular Grafts
Source: Biomater Res. 2025 Oct 17;29:0241. doi: 10.34133/bmr.0241 (PMC12531493; doi:10.34133/bmr.0241)
Supplement: Supplementary 1 — Figs. S1 to S5 [file bmr.0241.f1.docx]

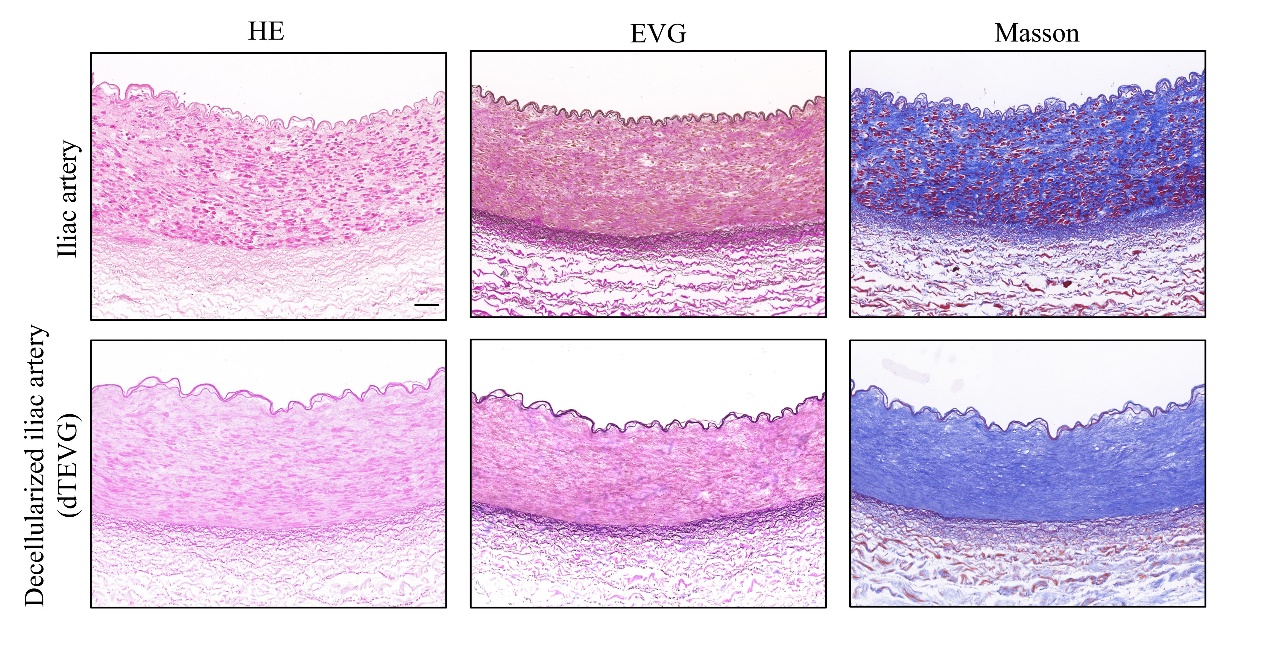
Supplementary figure 1 HE staining, EVG staining, and Masson staining results of human iliac artery and decellularized iliac artery (dTEVG). Scale bar, 100μm.

Supplementary figure
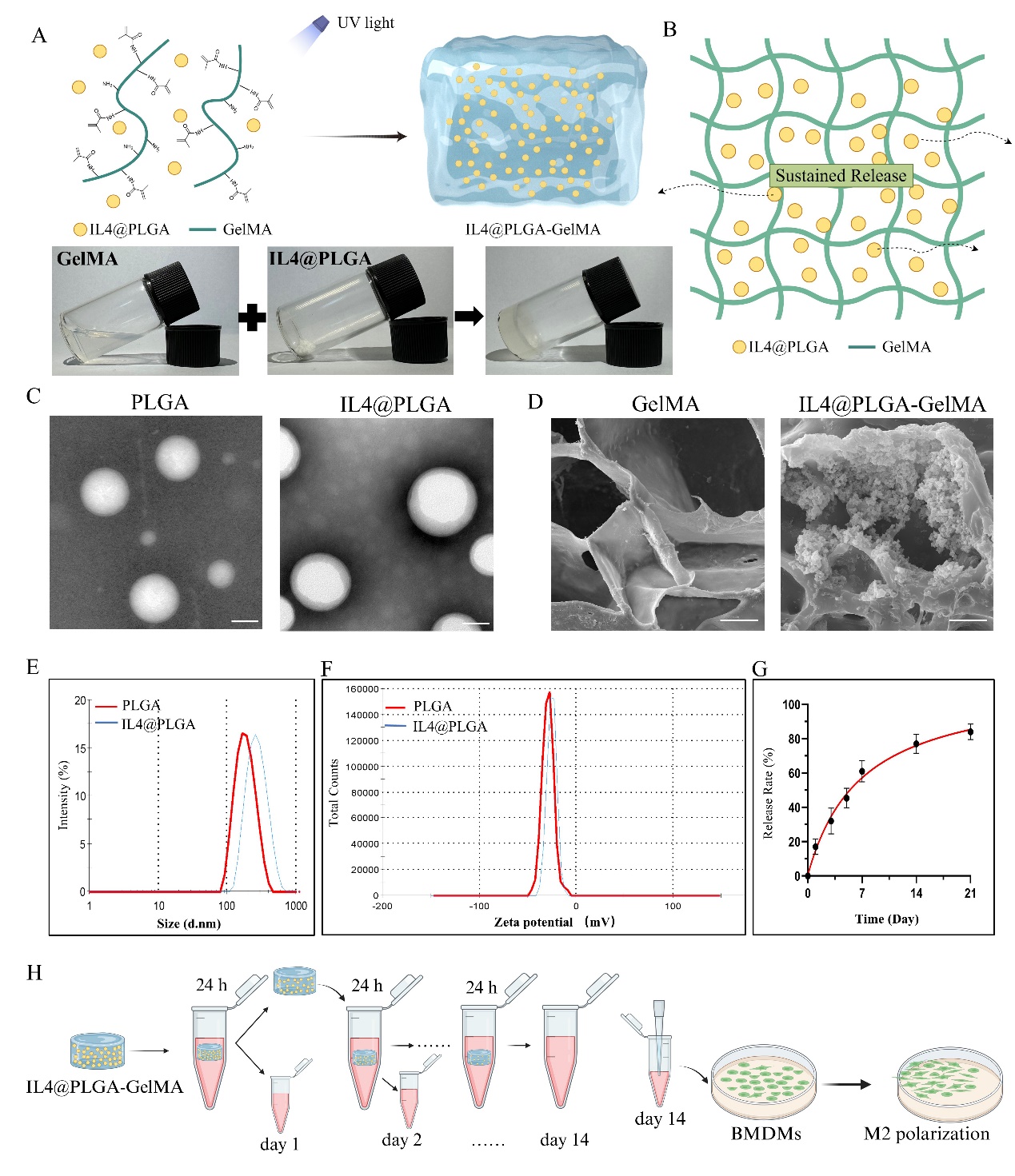
2 Preparation and characterization of IL4 NP-GelMA hydrogel. (A) Schematic illustration of the preparation of IL4@PLGA-GelMA hydrogel. (B) Schematic illustration of the drug stability and sustained release capability of IL4@PLGA-GelMA hydrogel. (C) TEM images of IL4@PLGA nanoparticles. Scale bar, 50 nm. (D) SEM images of IL4@PLGA-GelMA hydrogel. Scale bar, 10μm. (E) Particle size analysis of IL4@PLGA nanoparticles. (F) Zeta potential measurement of IL4@PLGA nanoparticles. (G) Stable and sustained release of IL4 from IL4@PLGA-GelMA hydrogel. (H) Schematic illustration of macrophage polarization towards M2 phenotype using hydrogel that has been continuously released for 14 days.


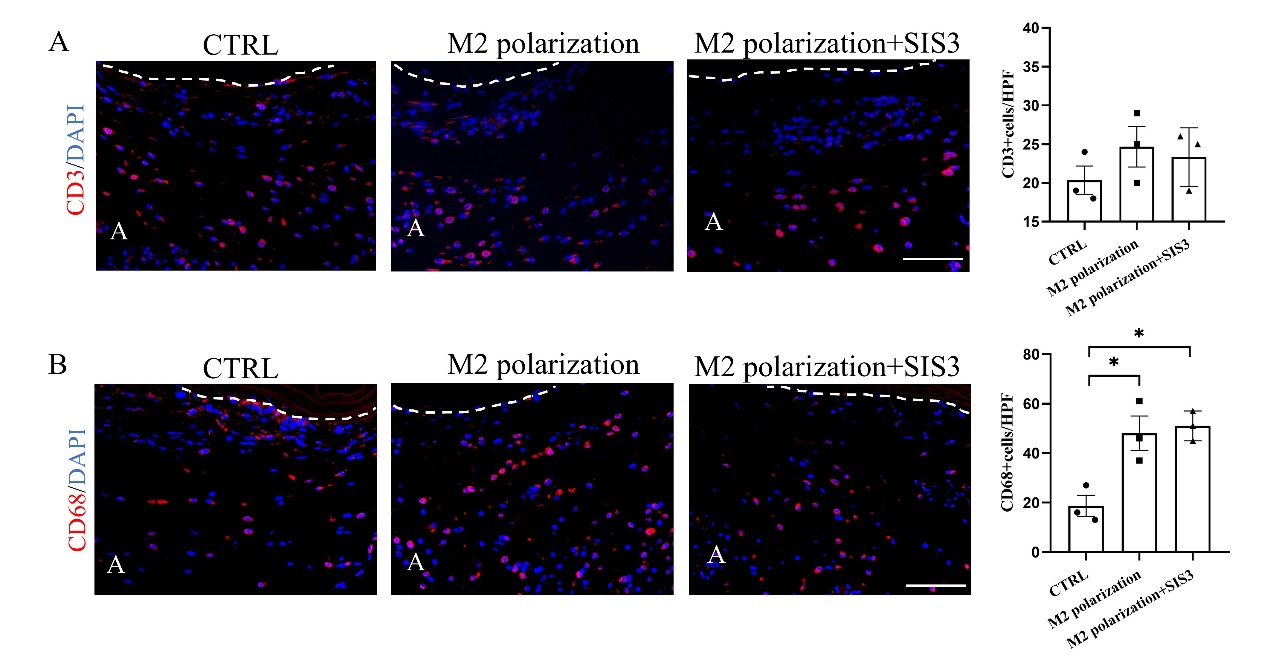


Supplementary figure 3 Infiltration of CD3-positive T cells and CD68-positive macrophages in the adventitia of dTEVG before and after enhanced M2 macrophage infiltration. (A) Enhanced M2 macrophage infiltration and SIS3 intervention did not affect the number of infiltrating CD3-positive T cells. Scale bar, 50μm. (B) Enhanced M2 macrophage infiltration increased the number of CD68-positive macrophages, while SIS3 intervention had no effect on macrophage infiltration. Scale bar, 50μm.


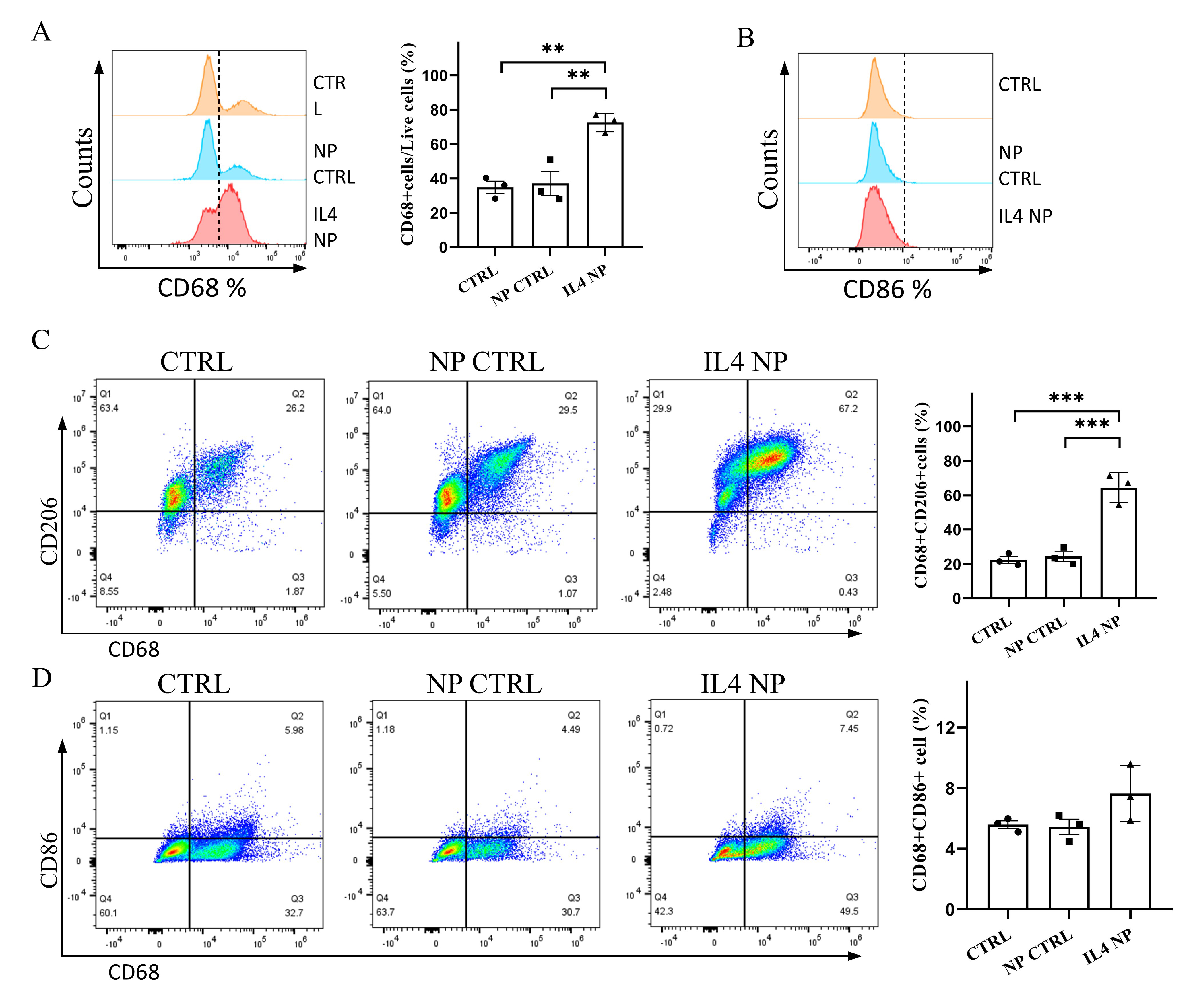


Supplementary figure 4 Flow cytometry of in vivo dTEVG samples after 21 days implantation. (A) The proportion of CD68⁺ cells increase after IL4 NP-GelMA hydrogel intervention (n=3). (B) The proportion of CD86-positive cells showed no significant change before and after intervention (n = 3). (C) The proportion of CD68⁺CD206⁺ cells significantly increased after IL4 NP-GelMA hydrogel intervention (n = 3). (D) The proportion of CD68⁺CD86⁺ cells remained unchanged after IL4 NP-GelMA hydrogel intervention, and CD86-positive cells accounted for only a small fraction of the population (n = 3).


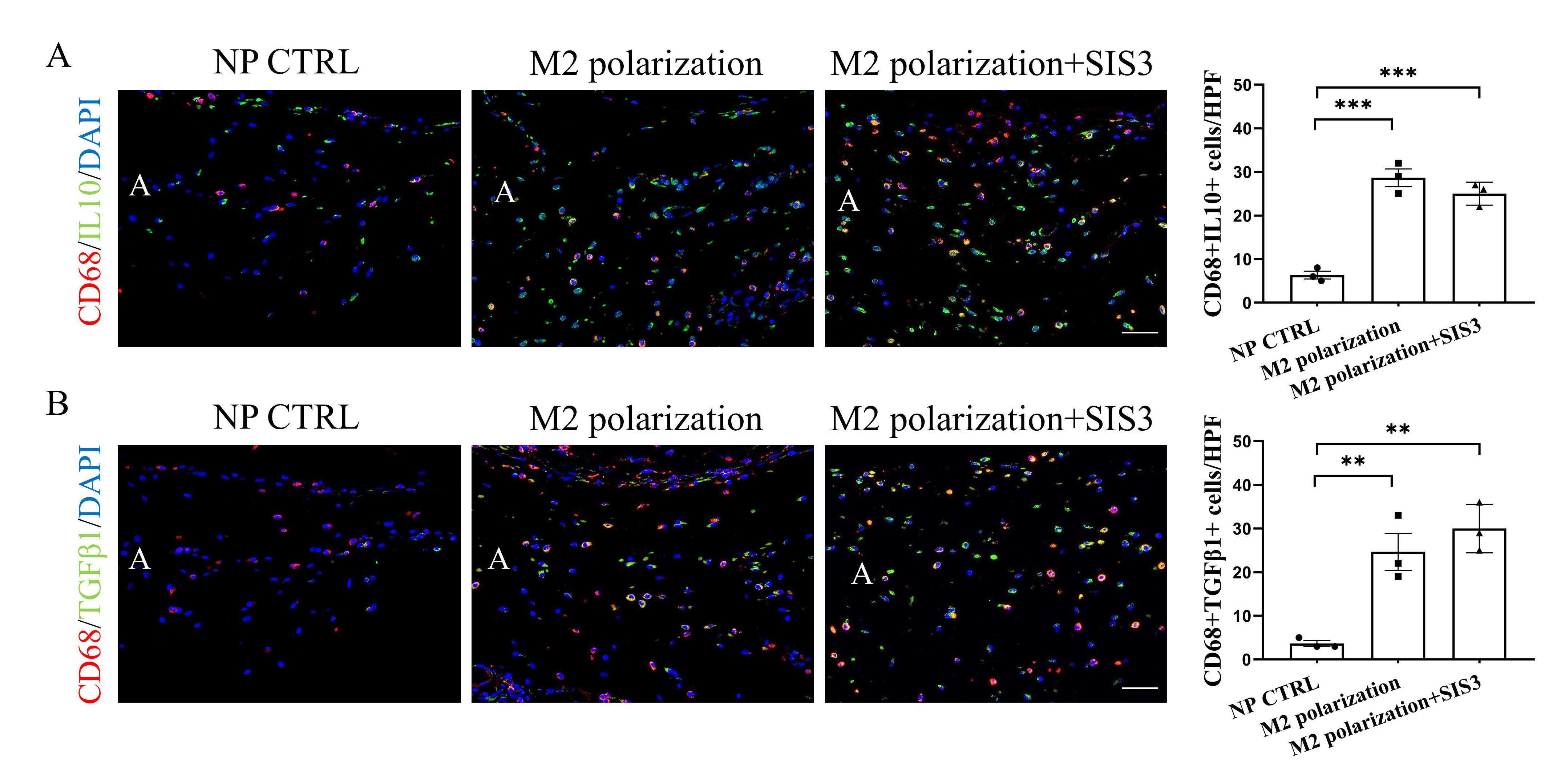
Supplementary figure 5. Inhibition of the Smad3 phosphorylation did not affect M2 macrophage infiltration and function. (A) IF of CD68 and IL10 of dTEVG in the NP CTRL, M2 macrophage polarization (IL4 NP), and M2 macrophage polarization+SIS3 groups. Scale bar, 50μm. (B) IF of CD68 and TGFβ1 of dTEVG in the NP CTRL, M2 macrophage polarization (IL4 NP), and M2 macrophage polarization+SIS3 groups. Scale bar, 50μm. Scale bar, 50μm. A represents the adventitia.
